# Analysing the impact of contextual segments on the overall rating in multi-criteria recommender systems

**DOI:** 10.1186/s40537-023-00690-y

**Published:** 2023-02-05

**Authors:** Chinta Venkata Murali Krishna, G. Appa Rao, S. Anuradha

**Affiliations:** Department of CSE, GITAM (Deemed To Be University), Vishakapatnam, A.P India

**Keywords:** Recommender system, Collaborative filtering, Hotel classes, Trip types and backward elimination

## Abstract

Depending on the RMSE and sites sharing travel details, enormous reviews have been posted day by day. In order to recognize potential target customers in a quick and effective manner, hotels are necessary to establish a customer recommender system. The data adopted in this study was rendered by the Trip Advisor which permits the customers to rate the hotel on the basis of six criteria such as, Service, Sleep Quality, Value, Location, Cleanliness and Room. This study suggest the multi-criteria recommender system to analyse the impact of contextual segments on the overall rating based on trip type and hotel classes. In this research we have introduced item-item collaborative filtering approach. Here, the adjusted cosine similarity measure is applied to identify the missing value for context in the dataset. For the selection of significant contexts the backward elimination with multi regression algorithm is introduced. The multi-collinearity among predictors is examined on the basis of Variance Inflation Factor (V.I.F). In the experimental scenario, the results are rendered based on hotel class and trip type. The performance of the multiregression model is evaluated by the statistical measures such as R-square, MAE, MSE and RMSE. Along with this, the ANOVA study is conducted for different hotel classes and trip types under 2, 3, 4 and 5 star hotel classes.

## Introduction

The tourism industry plays a major role for the growth of country’s economy. In order to scatter the tourism information the internet plays a major role in most of the countries. Currently everyone wishes to energize themselves in the vacation by visiting the locations all around the globe in the categories of middle and upper sections of users [1]. Once in a year the users plan their vacations due to an increase in socioeconomic factors. To fulfil their aspirations online travel platform is one of the great opportunity. To resolve the information overload issue, the recommender system was introduced to help the users by analysing the user preference information [2]. Based on recommendation approach the recommender systems can be categorised in to content-based, collaborative filtering (knowledge-based), and hybrid [3, 4]. The content based recommendation only believe on users past preferences to construct their profile and select suggested items. To identify the candidate items, the collaborative filtering approaches examine the behaviours of similar users [5].

Recommender systems are extensively utilized in most of the multimedia RMSE in order to improve personalization capabilities by focusing media products to the corresponding customers [6, 7]. Due to excess recommender systems, many customers receive non-detailed, non-personalized recommendation services such as old spam emails. Hence from the hotel’s opinion, it is essential to precisely recognize and increase the customer visit. When considering the customer’s opinion, they require recommendations only from the suitable hotels instead of gaining promotions from various hotels [8, 9]. Hence, the promotion of hotel can be done efficiently through personalized recommendation with the available customers at the hotel. The order rate of the customer, credibility and recognition of the hotel can also be maximized. The shopping cites inspire users to write review text for the purchased products. The reviews given by the previous reviewers are a useful understanding of the users and it enhances the recommendation ability of a website [10, 11].

Collaborative filtering (CF) is one of the most frequently used method in various fields to recommend items. In CF approach the recommendation had been done based on users and items [13]. The user to user or item to item similarity can be evaluated based on ratings. Most frequently CF technique utilizes single-criterion rating for recommendation however due to some limitations the users failed to grasp accurate recommendation results. Therefore to overcome this issues and to improve the recommendation accuracy multi-criteria recommender systems (MCRSs) have been progressed [14, 15]. The user opinion or preference regarding an item can be recommended by recommendation systems. In tourism industry, the most important and widespread online activity is the information searched by the travellers. Many tourism studies specify that most of the users undergo trip planning on the basis of information present in the online tourist RMSE. The user information plays a vital role in online travellers’ decision making. Most of the travellers plan their trip based on the reviews generated by the previous users. Regarding the accommodation experience in hotels, the users can expose their views and ratings through one of the leading travel opinion platform called Trip Advisor.

Hence, CF based item-item filtering along with the multiple regression backward elimination based multi recommendation system is introduced in this research. The goal behind the development of the recommendation system is to provide the more accurate recommendation based on the user preferences. Several approaches were devised for recommending the user preference based on the multiple criteria; still the computational overhead and inaccurate recommendation prevails. Thus, for obtaining the accurate recommendation by considering the multi-criteria based on CF is proposed. The major contributions of the research are:

- *Proposed Filtering technique*: The pre-processing of the input data is employed by two various factors like missing values imputation based on adjusted cosine similarity and filtering. Here, item-item collaborative filtering (CF) is proposed for filtering the significant context based on the user preference, which helps to enhance the prediction rating.
- *Proposed Multiple Regression Backward Elimination*: The multi-recommendation is employed based on the multiple regression criteria, in which the backward elimination is employed for the elimination of the inappropriate features based on the significant level. Here, the inappropriate feature elimination helps to minimize the computation overhead and enhance the accuracy of recommendation.
- *Analysis*: The recommendation of the hotel classes and trip type based on the significant context is analyzed based on R-square, MAE, MSE and RMSE to depict the superiority of the introduced recommendation system.

The highlights of proposed approach are:

- Multi-criteria recommendation system based on contextual segments.
- Proposed Item-item collaborative filtering for filtering the significant context based on the user preference.
- The multi-recommendation is introduced based on the multiple regression criteria, in which the backward elimination is employed for the elimination of the inappropriate features based on the significant level.
- The analysis based on R-square, MAE, MSE and RMSE evaluation measures to depict the superiority of the introduced recommendation system.

The organization of the paper is described as follows: “Related work” Section describes the related work. “Proposed Methodology” Section describes the proposed methodology. Finally the results and conclusion parts are described in “Results and discussions” Section and “Conclusion” Section.

## Related work

The review of conventional methods of multi criteria recommendation systems are: Hong and Jung [16] had proposed multi-criteria tensor model for tourism recommender systems. Several tourism recommender systems have been proposed and those systems reflect the multi-criteria ratings and the cultural differences. Higher Order Singular.

Value Decomposition (HOSVD) was utilised to predict missing values of the model. The author in [16] had developed two single tensor models and the tensor model is illustrated with four dimensions such as, user, items, multi-criteria rating (food, service, price and overall) and cultural groups. The integer value or the rating score ranges from 1 to 5 and it denotes the most positive and negative reviews. In addition to this, tensor factorization had introduced to predict the unobserved users’ preferences for restaurants. In the experimental section the performance measure of root mean square error (RMSE) and mean absolute error (MAE) are evaluated.

Nilashi et al. [17] had proposed multi criteria collaborative filtering approach for eco-friendly hotels recommendation. In this research the author had developed soft computing model by the integration of machine learning model to identify best matching eco-friendly hotels with the aid of several quality factors in TripAdvisor. Here, both the dimensionality reduction and prediction had been done to progress the scalability of the model. For dimensionality reduction, theHOSVD model was introduced. In addition to this the clustering of data in Trip Advisor dataset had been executed by Self-Organizing Map (SOM). The next stage is feature selection and it is an essential stage. This stage has been executed by decision trees technique. Adaptive Neuro-Fuzzy Inference Systems (ANFIS) model was intended for an accurate prediction.In the experimental section, the predictive model was measured by two statistical performance such as, RMSE and adjusted coefficient of determination. Along with this the recommendation quality had been proved by evaluating the precision, recall and f-measure.

Quasi Shambour [18] had developed deep learning approach for multi criteria recommendation system. Compared to single criterion recommender system, multi criteria recommended system attains more accurate outcome. Deep auto encoder model was introduced for multi-criteria recommender systems. The author built auto encoder based multi-criteria recommendation algorithm (AEMC) in which it employs deep feed forward neural network. In the experimental section the TripAdvisor multi-criteria datasets is used and compared with existing methods to prove the efficacy of the deep learning recommendation model. The prediction performance is evaluated by MAE and RMSE statistical metrics and also it yield an outcome of 0.64 and 0.72 consecutively.

Nassar [19] had proposed hybrid deep multi criteria model for recommender system and the deep learning models are the achievable remark in many fields. In this research the model was insisted with two major stages. In the first stage, the prediction had been done based on user ID and item ID. In the next stage, the prediction had been done based on deep neural network model. Five-fold cross validation test was conducted and the performance metrics of MAE, recall, precision and f-measure are evaluated.

Sagar et al. [20] had proposed collaborative and regression model for travel recommender system based on social media reviews due to COVID-19 pandemic. To examine the final score and find out the guest type, and also to replace the missing values, the collaborative filtering approach is introduced. In this research, the author had analysed only the Asian continent user opinion. Krishna et al. [21] had analyse the context with high significant of user with regression model. Here, the author had collected reviews from most popular tourist location all around the globe i.e. Singapore city from different star hotels. In the experimental scenario the statistical tests, co-relation between the users and ANOVA test are conducted.

Zhuang and Kim [22] had proposed Bidirectional Encoder Representations from Transformers (BERT) based multi criteria recommendation model for hotel promotion management. This study introduced BERT recommendation model to predict six criteria ratings. The proposed model is insisted with three stages namely data collection, BERT fine tuning and multi-criteria recommendation. In the experimental scenario the evaluation metrics of Hit Ratio (HR) and Normalized Discounted Cumulative Gain (NDCG) are evaluated. Singh et al. [23] had proposed item based collaborative filtering technique for enhancing recommendation using Bhattacharyya coefficients. Here, the data processing is emphasised by similarity metrics. In the experimental scenario the performance measure of RMSE and MAE are evaluated.

Samad [1] had introduced supervised and unsupervised machine learning model for analysing the customers’ online reviews. Author had introduced fuzzy rule machine learning model and clustering approach for recommendation. The intrusion of clustering technique will boost up the scalability and accuracy of the recommendation system. Self-Organizing Map (SOM) approach was introduced to cluster data in the Trip Advisor. In the experimental section the performance measure of MAE, precision and f measure are evaluated. From the surveys taken there is a research gap when the user’s preferences and priorities are insufficient. So in this research the multiple regression backward elimination with item-item collaborative filtering approach is introduced to identify the significant context.

Designing the personalized recommendation method which is useful for the users of IoT services was mandatory, such techniques needs to enhance the user experience. For such, an algorithm which combines the trusted relevance with matrix factorization was introduced by Li, W., et al. in [24]. An effective trust model that carefully integrates the social information of each user into recommendation algorithm for the recommendation based on user preferences. For that, initially the trust relationship in direct or indirect manner was considered to introduce the concentric hierarchical architecture related to social network. Then, the matrix factorization based recommendation algorithm was introduced which integrates most trust information within it. Finally, the trust and similar interest factors were comprehensively considered for developing trust relevance. This architecture achieved better prediction accuracy.

Two different aspects were considered as a major challenging issue for traditional recommendation algorithms they were, achieving high QoS parameters during recommendation and managing historical QoS data To overcome such issues, the LSH (Locality-Sensitive Hashing) and the location information of user/service were considered by Lin, W., et al. in [25]. These information were used for the location-aware recommendation framework which also enhances the privacy of the data. WS-DREAM dataset was used to prove the efficiency of this architecture.

Thus, the review of the prior recommendation systems has faced the challenges like:

- The failure in considering the significant attribute limits the performance of the model, which provides the inaccurate recommendation [16].
- The recommendation of the better hotels with eco-friendly characteristics devised by [17] fails to reduce the dimension of the data that elevates the computation overhead. The utilization of the significant attribute selection has the capability of reducing the computation complexity.
- The deep learning based approach introduced by [18] has the capability of getting the inaccurate outcome due to the minimal data utilized for training the model that limits the generalization capability.
- Content based recommendation system devised by [21] recommends the trip type based on the user preference; still, the scalability of the method is challenging due to the requirement of information updation for the new preference.

Thus, the challenges like inaccurate recommendation along with the enhanced computation overhead limit the performance of the traditional methods. The utilization of the CF filtering along with the multiple regression backward elimination enhances the recommendation accuracy with minimal computation complexity.

## Proposed methodology

**Fig. 1:**
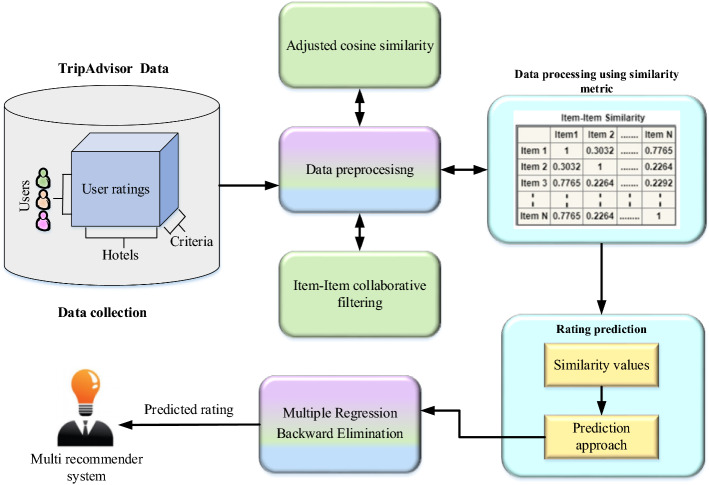
Global architecture of the proposed model

Enormous benefits are achieved in the human society on account of digital technology and social media. The traveller platform is rendered by the Trip Advisor who proceeds user generated content to share the opinions with respect to different aspects of hotel. In tourism domain, recommendation agents perform a significant role for hotel recommendations. In this study the work is processed under three stages namely data extraction, data pre-processing and rating prediction. The first stage is data extraction and in this stage the data are extracted from the Trip Advisor. Here, the data pre-processing is executed by item-item collaborative filtering approach. In addition to this the similarity is measured by the adjusted cosine similarity metrics. The final stage is prediction. In this stage based on multi regression backward elimination approach is introduced to analyse the impact on contextual segments. Here, the backward elimination is introduced to discard the irrelevant context and based on remaining context the prediction takes place. Finally the performance measure of MSE, RMSE and MAE and also the ANOVA test are conducted and evaluated. The global architecture of the proposed model is shown in (Fig. 1).

### Data pre-processing

The essential aspect of most recommendation systems is that each recommendation influences what is learned about the users and items, which decides the promising accuracy of future recommendations. The recommendation system insists on two approaches, namely content and collaborative filtering. Collaborative filtering (CF) is based on observed user preferences. Usually, the random value is contemplated to identify the nearby neighbor from the item-item similarity matrix. However, the deliberation of random value is not a rational approach since different items may have different values. Another challenging issue in collaborative filtering is sparsity in the dataset.

So in this research, instead of using random value, the adjusted cosine similarity measure is applied in the item-item CF approach. If computing the similarity between two items, initially, the users who have rated for both the items are isolated. After pinpointing the users, the similarity measure is applied.

There are several measures to calculate the similarity between two items, and in this research, the adjusted cosine similarity measure is utilized. The primitive difference between user and item-based CF is, in user-based CF, the similarity is measured based on matrix rows, and in item-based CF, the similarity is measured based on matrix columns. Similarity computation using traditional cosine based approach for item-based case has shown major demerit, i.e. it fails to account the rating scale exist between various users. This demerit is overcome by adjusted cosine similarity by subtracting the particular user average from the several co-rated pairs. The similarity between the two items ($n$ and $m$) in the item-item CF approach is calculated based on the below equation. The similarity measure between item $n$ and item $m$ are found to be maximum.

1 $$Sim\left( {n,m} \right) = \frac{{\sum\nolimits_{u \in U} {\left( {R_{u,n} - R_{u} } \right)\left( {R_{u,m} - R_{u} } \right)} }}{{\sqrt[2]{{\sum\nolimits_{u \in U} {\left( {R_{u,n} - R_{u} } \right)^{2} } }}\sqrt[2]{{\sum\nolimits_{u \in U} {\left( {R_{u,m} - R_{u} } \right)^{2} } }}}}$$

Here, $R_{u}$ represents the average of user’s ratings.

Usually, the similarity can be computed in several ways, such as user ratings, product descriptions, and co-occurrence of the items of the product purchased in the past. C1, C2, C3, C4, C5, and C6 are the contexts named Cleanliness, Location, Value, Rooms, Service, and Sleep-Quality. The general item-item collaborative filtering is shown in Algorithm 1.
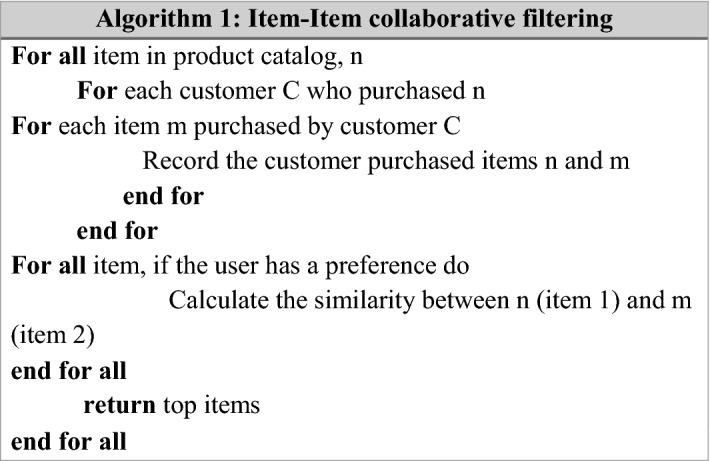


Algorithm 1 in this research intended a context-context collaborative filtering algorithm to find similar contexts. Here, similar contexts can be computed via adjusted cosine similarity measure. After applying this similarity measure, address the difference in rating scale between different users. The proposed context-context collaborative filtering algorithm is shown in Algorithm 2.
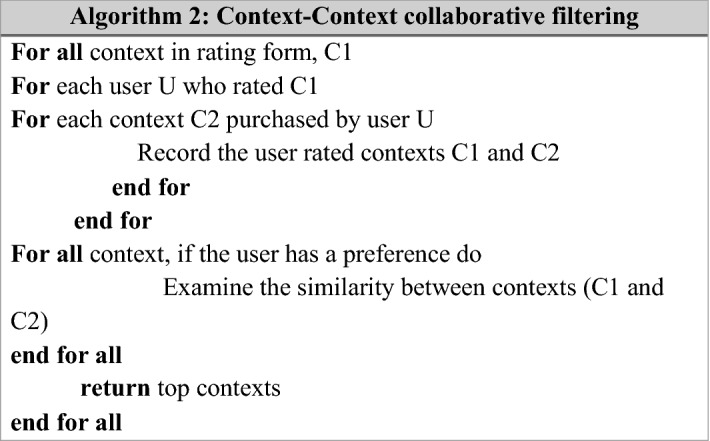


### Regression-based multi-criteria recommendation

Regression techniques are easy for processing as it is designed using the basic statistical principles. It takes less time to achieve best result. Moreover, the error attained during processing is also less. Due to this merits, the proposed architecture has introduced regression techniques for recommendation. Finally, the evaluated results also indicate that the proposed regression model has attained better performance than other regression models.

Multiple Regression Backward Elimination (MRBE) algorithm is introduced to identify the significant contexts, which have a high impact on overall rating. Backward elimination is a feature selection model that first eliminates the least important variables and leaves only the major essential variables in the model. In the regression model, all the variables are initially tested with a significance level of 0.05. If the p-value of the feature or context is greater than the significance value, $p > 0.05$, the elimination takes place. The same process gets repeated until all features become significant, $p < 0.05$. Finally, a set of features are defined, and this method increases the training time, diminish the complexity and improves performance.[12]

Multiple regression is a statistical technique used to explore the relationship between two or more variables. The multiple regression model with independent variables $\left( p \right)$ and size $\left( n \right)$ is represented by matrix notation, and it is given below:

2 $$y = \beta_{0} + \beta_{1} X_{1} + ... + \beta_{w} X_{w} + \xi$$

3 $$y = X\beta + \xi$$

Here,$y$ describes the dependent variable, $X$ describes the combination of $n \times p$ design matrix of independent variables. $\xi$ Symbolizes the residual terms or error vector of the regression model with the identity matrix $I$, $w$ signifies the number of observations or features and $\beta_{i}$ describes the regression coefficient or parameter of the model.

4 $$Y = \left[ {\begin{array}{*{20}c} {y_{1} } \\ {y_{2} } \\ \vdots \\ {y_{n} } \\ \end{array} } \right]\,\,\,\,\,X = \left[ {\begin{array}{*{20}c} 1 & {x_{11} } & \cdots & {x_{1p} } \\ 1 & {x_{12} } & \cdots & {x_{2p} } \\ \vdots & \vdots & \ddots & \vdots \\ 1 & {x_{n1} } & \cdots & {x_{np} } \\ \end{array} } \right]\,\,\,\,\,\beta = \left[ {\begin{array}{*{20}c} {\beta_{0} } \\ {\beta_{1} } \\ \vdots \\ {\beta_{{}} } \\ \end{array} } \right]\,\,\,\,\,\xi = \left[ {\begin{array}{*{20}c} {\xi_{1} } \\ {\xi_{2} } \\ \vdots \\ {\xi_{n} } \\ \end{array} } \right]$$

The cost function is emphasized to train the multiple regression model expressed in Eq. (1) to minimize the difference between the observed or true values and the fitted or predicted values. Root mean square error is the cost function used in this study, and it is expressed below:

5 $$RMSE = \sqrt {\frac{1}{n}\sum\limits_{j = 1}^{n} {\left( {y_{j} - \hat{y}_{j} } \right)^{2} } }$$

Here, $n$ defines the number of data points, $y_{j}$ and $\hat{y}_{j}$ describes the true and predicted values. The utilization of $R^{2}$ value or coefficient determines how well the predictor values fit the model and is expressed by Eq. (6).

6 $$R^{2} = 1 - \frac{{SS_{regression} }}{{SS_{total} }}$$

Here, $SS_{regression}$ and $SS_{total}$ sum of squares of the regression and a total number of squares. In linear regression, the use of $R^{2}$ is perfectly acceptable. When it comes to multiple regression, there will be slight variations in the formula due to the addition of the number of independent variables. Therefore, the $R^{2}$ value makes a significant difference in the multiple regression by added variables. The mathematical expression is defined below:

7 $$Adjusted\,R^{2} = 1 - \left( {1 - R^{2} } \right)\left( {\frac{n - 1}{{n - \left( {w + 1} \right)}}} \right)$$

$w$, signifies the number of predictors in the regression equation. In addition, the ordinary least squares (OLS) regression model is constructed to diminish the residual sum of squares, and the mathematical expression is given below:

8 $$RSS\left( \beta \right) = \left( {y - X\beta } \right)^{T} \left( {y - X\beta } \right)$$

Identifying the multi-collinearity between the independent variable is essential to identify the significant context. Here, the overall rating is selected as the dependent variable and the cleanliness, location, value, rooms, service, sleep quality are selected as the independent variables. In which the multi-collinearity can be checked among the independent variables to variance impact factor (VIF), and the mathematical expression is defined below:

9 $$VIF\left( {\beta_{i} } \right) = \frac{1}{{1 - R_{i}^{2} }}$$

Here, $R_{i}^{2}$ specifies the squared coefficient of the regression model. If the independent variables are uncorrelated, then $R_{i}^{2} = 0$ and in case of exact collinearity $R_{i}^{2} = 1$. Therefore, $VIF\left( {\beta_{i} } \right)$ tends to be one and infinity. The algorithm for multiple regression backward elimination is shown in Algorithm 3. Here, the backward algorithm is applied to identify the significant contexts for each hotel class and trip type. Also, this research mainly focused on the best independent variables to forecast the Predicted overall rating. The major steps of the backward elimination are shown below:


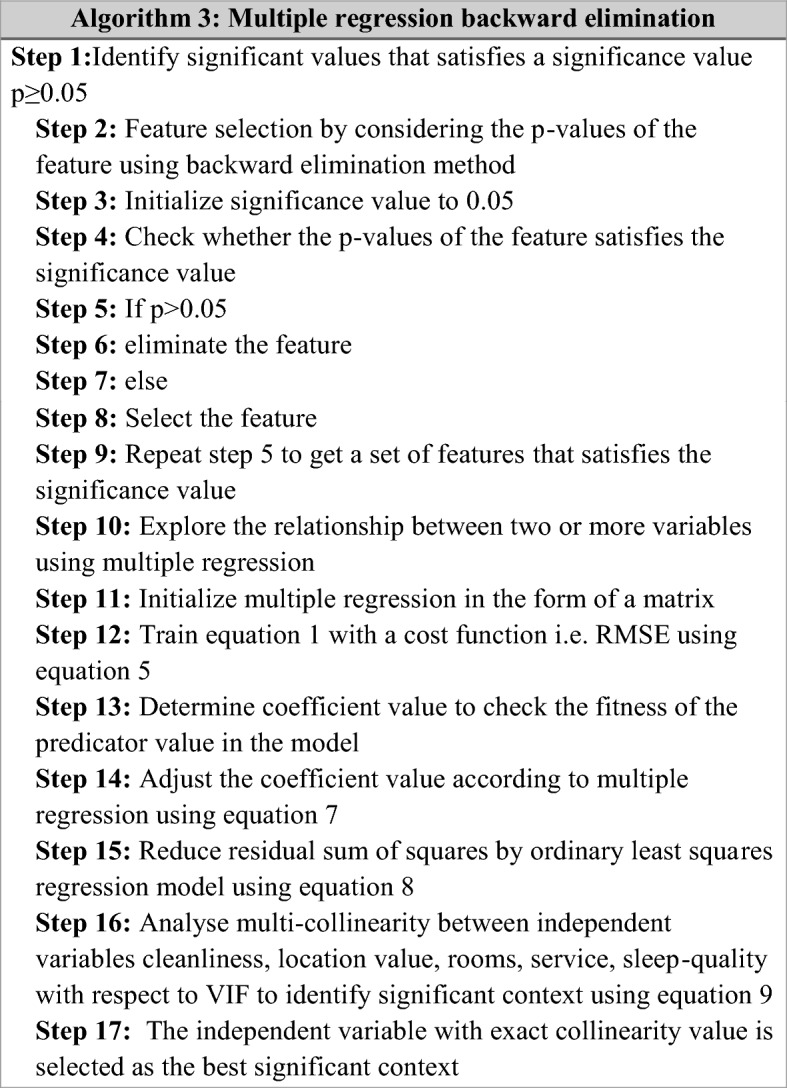


## Results and discussions

To analyse the effectiveness of the proposed model, several experiments were conducted on Trip Advisor datasets provided by the Trip Advisor website (www.tripadvisor.com). Trip Advisor is the most important world’s largest successful social network. On this website, the users can prefer a hotel due to some aspects such as cleanliness, location, value, rooms, service and sleep quality. The dataset was extracted from Trip Advisor through web scrapping using beautiful Soup. 93 tourism cities across the world from different continents such as Asia, Europe, Australia, Africa, North America, and South America are chosen according to the tourism rankings given by Master Card and Visa. Totally, 60,215 records were collected from 2500 hotels across 93 cities. In this research, the analysis is carried out in two manners. The data set is classified into two categories: hotel class (two, three, four and five stars) and trip type (Business, Family, Friends, Couple, Solo, N.A (Not mention any trip-type)). Therefore, the results are analysed accordingly. For each Hotel class and Trip-Type significant contexts were identified upon overall rating on the continental, county, and city wise to identify the user opinions on hotel stays.

### Analysis of performance measures based on different hotel classes and trip types

To evaluate the performance of the multi regression model, the statistical accuracy metrics are evaluated. The metrics used for evaluation are R-square, mean absolute error (MAE), mean square error (MSE) and root mean square error (RMSE). The measure of error between paired observations is termed MAE. RMSE is a standard way to compute the error of a model. The metrics MSE measures the average of the square of error. The mathematical expression of MAE, MSE and RMSE is defined below:

10 $$MAE = \frac{1}{N}\sum\limits_{u,i}^{N} {\left| {p_{u,i} - r_{u,i} } \right|}$$

11 $$RMSE = \sqrt {\frac{1}{N}\sum\limits_{u,i}^{N} {\left( {p_{u,i} - r_{u,i} } \right)^{2} } }$$

12 $$MSE = \sum\limits_{u,i}^{N} {\left( {p_{u,i} - r_{u,i} } \right)^{2} }$$

Here, $N$ specifies the total number of ratings, $p_{u,i}$ describes the predicted rating of user $u$ given on item $i$ and $r_{u,i}$ resembles the actual rating. Therefore, the statistical metrics are analysed for multi regression models such as decision tree regression (DTR), linear regression (LR), random forest regression (RF) and support vector regression (SVR) are analysed for different hotels classes and trip types. (Table 1) shows the overall accuracy of the initial and predicted results based on different hotel classes, namely, 5, 4, 3 and 2 stars consecutively. Tables (2, 3, 4, 5) shows the accuracy analysis of regression models based on trip types (business, couple, friend, family and solo) under hotel classes. The baseline details about the regression models are discussed below:

**Table 1:** Hyperparameters of different methods

Method	Hyperparameter
DTR	Maximum depth = 10 Criterion–Gini index
LR	Weight constraint–5 Regularization penalty [0, 1] Dropout rate–0.075
RF	Maximum depth–8 Number of estimators–150 Max sample split–15 Min-sample-leaf–10
SVR	Kernel–radial basis Penalty C–1.0 Log (gamma) =− 3

**Table 2:** Performance analysis for different hotel classes (Initial and predicted results)

Results	Hotel class	Metrics	Regression models (Initial)			
			DTR	LR	RF	SVR
Initial	5	R-Square	0.238710167	0.489928223	0.489778096	0.521418161
		MAE	0.599889502	0.604654344	0.532280348	0.520965902
		MSE	1.156266891	0.774710343	0.77493836	0.726882603
		RMS	1.075298512	0.880176314	0.880305833	0.852574104
	4	R-Square	0.43602592	0.5587822	0.569708085	0.561356309
		MAE	0.45501558	0.482444483	0.428675671	0.423126638
		MSE	0.637938448	0.499082872	0.486724073	0.496171172
		RMS	0.798710491	0.706457976	0.697656128	0.704394188
	3	R-Square	0.543701834	0.606134681	0.639348622	0.630621703
		MAE	0.419253732	0.45881797	0.402924485	0.38546005
		MSE	0.49490954	0.427193705	0.391169242	0.400634622
		RMS	0.703498074	0.65360057	0.625435242	0.632957046
	2	R-Square	0.584992986	0.673279484	0.686638412	0.697695504
		MAE	0.400416941	0.429079191	0.379938307	0.358382134
		MSE	0.460809496	0.362779209	0.347945915	0.335668502
		RMS	0.678829504	0.602311555	0.589869405	0.579369055
Predicted	5	R-Square	0.987971653	1.0	0.996103707	0.992352129
		MAE	0.035758734	1.21503E−15	0.021177633	0.068714838
		MSE	0.008977325	2.18013E−30	0.002907988	0.005707968
		RMS	0.094748744	1.47653E−15	0.053925761	0.075551092
	4	R-Square	0.994806161	1.0	0.998082428	0.99052232
		MAE	0.015350923	2.6666E−15	0.010184623	0.070867521
		MSE	0.003365271	1.38434E−29	0.001242463	0.006140923
		RMS	0.05801096	3.72068E−15	0.035248582	0.078364039
	3	R-Square	0.996162427	1.0	0.998118141	0.98985513
		MAE	0.011227245	9.01114E−16	0.00845773	0.071785537
		MSE	0.002402652	1.66537E−30	0.001178206	0.006351565
		RMS	0.049016852	1.29049E−15	0.034325007	0.079696703
	2	R-Square	0.993487236	1.0	0.996340491	0.987468051
		MAE	0.018764374	1.03616E−15	0.014411304	0.076915324
		MSE	0.004671013	1.9147E−30	0.002624633	0.008988026
		RMS	0.068344809	1.38373E−15	0.051231171	0.0948052

**Table 3:** Performance analysis on 5 star hotel class based on trip type (Initial and predicted results)

	Hotel class: 5					
	Trip type	Metrics	Regression models			
			DTR	LR	RF	SVR
Initial	Business	R-Square	0.330098467	0.584736234	0.544640684	0.604195933
		MAE	0.605619383	0.581940599	0.533897951	0.501243912
		MSE	1.144838817	0.709671579	0.778193503	0.676415618
		RMS	1.069971409	0.842420073	0.882152766	0.822444903
	Couple	R-Square	0.218133957	0.476638312	0.461115074	0.519604393
		MAE	0.504565917	0.506479352	0.449567603	0.439952932
		MSE	0.969162141	0.648733039	0.667974871	0.595474428
		RMS	0.98446033	0.805439656	0.817297296	0.771669895
	Friends	R-Square	−0.24990781	0.313490934	0.273297993	0.311164439
		MAE	0.916470438	0.780166938	0.746868378	0.717472347
		MSE	2.15190259	1.181927679	1.251125818	1.185933087
		RMS	1.466936464	1.087164973	1.118537356	1.089005549
	Na	R-Square	0.034345766	0.24146114	0.453187809	0.372348188
		MAE	0.700060823	0.713014732	0.586768372	0.610872291
		MSE	1.376256805	1.081074604	0.779320354	0.894533517
		RMS	1.173139721	1.039747375	0.882791229	0.94579782
Predicted	Business	R-Square	0.977011612	0.977011612	0.977011612	0.977011612
		MAE	0.058746365	8.59395E−16	0.031914755	0.077447023
		MSE	0.021728744	1.19335E−30	0.005765376	0.008678885
		RMS	0.14740673	1.09241E−15	0.075930075	0.093160533
	Couple	R-Square	0.985430866	1.0	0.9919396	0.981434517
		MAE	0.032680946	6.67884E−16	0.025366234	0.084143538
		MSE	0.008208131	1.02359E−30	0.004541164	0.010459641
		RMS	0.090598738	1.01173E−15	0.067388157	0.102272388
	Family	R-Square	0.972981332	1.0	0.984517143	0.985366584
		MAE	0.055371583	6.27795E−16	0.037737825	0.082964182
		MSE	0.019848843	9.87318E−31	0.011374239	0.010750211
		RMS	0.140885923	9.93639E−16	0.106650079	0.103683226
	Friends	R-Square	0.931534276	1.0	0.978869909	0.989496157
		MAE	0.11308985	4.45759E−16	0.068180115	0.064613082
		MSE	0.043581077	3.64033E−31	0.013450119	0.006686102
		RMS	0.208760814	6.03351E−16	0.115974647	0.081768586
	Na	R-Square	0.872149309	1.0	0.963560265	0.976579223
		MAE	0.122244828	7.17179E−16	0.066346766	0.089176871
		MSE	0.057325331	7.32379E−31	0.016338745	0.010501342
		RMS	0.239427088	8.55791E−16	0.1278231	0.102476054

**Table 4:** Accuracy analysis on 4 star hotel class based on trip type (Initial and predicted results)

	Hotel class: 4					
	Trip type	Metrics	Regression models			
			DTR	LR	RF	SVR
Initial	Business	R-Square	0.500499663	0.571730015	0.622637838	0.60432398
		MAE	0.454416452	0.492318468	0.427498911	0.432185146
		MSE	0.62427322	0.535249854	0.471625491	0.494514066
		RMS	0.790109625	0.731607719	0.686749948	0.703216941
	Couple	R-Square	0.379408723	0.56261977	0.566966686	0.5821451
		MAE	0.407322959	0.428936758	0.37600497	0.360798776
		MSE	0.531212538	0.374387896	0.37066703	0.35767464
		RMS	0.728843288	0.61187245	0.608824302	0.598059061
	Family	R-Square	0.427375634	0.517426807	0.537317355	0.518186975
		MAE	0.417756143	0.467897336	0.402319383	0.409406005
		MSE	0.595395551	0.501763371	0.481081849	0.500972974
		RMS	0.771618786	0.708352575	0.693600641	0.707794443
	Friends	R-Square	0.231602701	0.464082448	0.470741684	0.473549516
		MAE	0.485983094	0.526916745	0.453298693	0.45965649
		MSE	0.844423055	0.588941602	0.581623498	0.578537857
		RMS	0.918924946	0.767425307	0.762642444	0.760616761
	Solo	R-Square	0.406590011	0.524378896	0.559923796	0.551848304
		MAE	0.472640739	0.491888024	0.439279924	0.436842432
		MSE	0.654771874	0.524802965	0.485582525	0.494493067
		RMS	0.809179754	0.724432857	0.696837517	0.70320201
Predicted	Business	R-Square	0.986917138	1.0	0.995109276	0.990896252
		MAE	0.030853037	1.33454E−15	0.020840004	0.071659678
		MSE	0.009975699	3.26819E−30	0.003729183	0.006941619
		RMS	0.09987842	1.80781E−15	0.061067038	0.083316381
	Couple	R-Square	0.992325901	1.0	0.99653063	0.984578646
		MAE	0.016069413	9.73099E−16	0.011468717	0.072643083
		MSE	0.003679316	1.88399E−30	0.001663376	0.007393707
		RMS	0.060657369	1.37259E−15	0.040784503	0.08598667
	Family	R-Square	0.992672864	1.0	0.996400982	0.986364859
		MAE	0.020320878	1.4341E−15	0.015203218	0.078010729
		MSE	0.004557864	3.67711E−30	0.002238779	0.008481775
		RMS	0.067511957	1.91758E−15	0.047315735	0.092096554
	Friends	R-Square	0.97854352	1.0	0.992613472	0.984379061
		MAE	0.039211831	1.79106E−15	0.026019507	0.080138649
		MSE	0.012690384	5.26391E−30	0.004368745	0.009238967
		RMS	0.112651606	2.29432E−15	0.066096481	0.096119547
	Solo	R-Square	0.971336701	1.0	0.99026373	0.985296617
		MAE	0.054406343	1.21503E−15	0.031120354	0.077679192
		MSE	0.017115719	2.18013E−30	0.00581382	0.008779833
		RMS	0.130827059	1.47653E−15	0.076248409	0.093700762

**Table 5:** Accuracy analysis on 3 star hotel class based on trip type (Initial and predicted results)

	Hotel class: 3					
	Trip type	Metrics	Regression models			
			DTR	LR	RF	SVR
Initial	Business	R-Square	0.494034631	0.680403331	0.643805012	0.664326333
		MAE	0.465347868	0.453325862	0.429376229	0.409318226
		MSE	0.612187811	0.386692839	0.430974615	0.406145045
		RMS	0.782424316	0.621846315	0.656486569	0.6372951
	Couple	R-Square	0.557112178	0.637619169	0.652403661	0.66072788
		MAE	0.370073741	0.41151151	0.360877671	0.34315076
		MSE	0.405376986	0.331688617	0.318156312	0.310537122
		RMS	0.636692222	0.575924142	0.564053466	0.557258577
	Family	R-Square	0.513912241	0.665043759	0.671508988	0.694180945
		MAE	0.417504338	0.430080134	0.382853486	0.369920292
		MSE	0.552125479	0.380461905	0.373118339	0.347366271
		RMS	0.743051464	0.616815941	0.610834134	0.589377868
	Friends	R-Square	0.345443636	0.547108351	0.501324814	0.512815694
		MAE	0.447834358	0.458106296	0.423223556	0.409417494
		MSE	0.616487519	0.426551577	0.469672354	0.458849781
		RMS	0.785167192	0.653109162	0.685326458	0.677384515
	Na	R-Square	0.424480129	0.531362074	0.564709185	0.57679988
		MAE	0.470872837	0.497619667	0.428460934	0.435608361
		MSE	0.582264244	0.474129777	0.440391879	0.428159496
		RMS	0.763062412	0.688570822	0.663620282	0.654338977
	Solo	R-Square	0.409096675	0.556488118	0.565203171	0.603410223
		MAE	0.468043902	0.486694355	0.432149131	0.402903409
		MSE	0.61475666	0.461415382	0.452348523	0.412599145
		RMS	0.784064194	0.67927563	0.672568601	0.642338808
Predicted	Business	R-Square	0.988710956	1.0	0.993934999	0.98985513
		MAE	0.032276306	7.99261E−16	0.023254645	0.071785537
		MSE	0.009410853	9.62679E−31	0.00505595	0.006351565
		RMS	0.097009551	9.81162E−16	0.071105202	0.079696703
	Couple	R-Square	0.991022773	1.0	0.996989662	0.983256503
		MAE	0.016769973	9.4321E−16	0.010718847	0.078230875
		MSE	0.005175668	1.54316E−30	0.001735559	0.00965318
		RMS	0.071942116	1.24224E−15	0.041660045	0.098250597
	Family	R-Square	0.993925877	1.0	0.997542518	0.985266241
		MAE	0.018471197	8.31279E−16	0.01310571	0.081516663
		MSE	0.004495411	1.19969E−30	0.001818764	0.01090434
		RMS	0.067047824	1.0953E−15	0.042646965	0.104423849
	Friends	R-Square	0.978614861	1.0	0.993251005	0.979149358
		MAE	0.026759625	1.01946E−15	0.018504015	0.085447037
		MSE	0.009787937	1.53945E−30	0.003089002	0.009543299
		RMS	0.098934003	1.24075E−15	0.055578791	0.097689809
	Na	R-Square	0.953731657	1.0	0.985717484	0.97915379
		MAE	0.050709028	7.42076E−16	0.029443623	0.08062522
		MSE	0.022985987	1.34379E−30	0.007095515	0.01035634
		RMS	0.151611302	1.15922E−15	0.08423488	0.101766105
	Solo	R-Square	0.981319065	1.0	0.989753786	0.976194522
		MAE	0.031211047	8.41058E−16	0.022538522	0.087649318
		MSE	0.009594515	1.4182E−30	0.005262449	0.012226478
		RMS	0.097951598	1.19088E−15	0.072542737	0.110573404

#### DTR

DTR [27] is a regression model which obtains the predicted output by mapping the input with the attributes. The interior node present in tree is represented for attributes and an arc is formed between the parent and child those who having the possible values related to that attributes. Initially, the tree construction begins with input set and root node. For each root, an attribute is assigned and then a set of values are assigned for each arcs and sub-nodes. Then, the values of each input set are divided, therefore the child node receives only a specific portion of input set that matches with the attribute value (value specified by each arc corresponding to child node). Till reaching last split, the process recursively happens for each child node.

#### LR

LR [28] is a statistical process which is used to determine the relationship between the independent variable X and dependent variable Y. For simple linear regression, the independent variable is fixed as 1 and more than one independent variable is represented for multi-linear regression. Linear regression is a process which aggregate the similarities and determine the overall ratings based on the weights of each criterion.

#### RF

RF [29] is an efficient method which works effectively for huge datasets. It can effectively performs the recommendation with available data without causing any deterioration in system performance. DT based integrated individual learners are included in RF. A subset of random training data are used for tree generation. After training each forest, the test rows are introduced to each forest. An output class is generated from each tree and the mode related to each classes are taken as output from the RF.

#### SVR

SVR [30] will provide maximum fit points in the hyperplane, hence the regression line value could be obtained very accurately, and meanwhile it provide only discrete values cannot be used for continuous prediction problems. By using SVR the error between real and predicted data will be very low by having the capability of fitting within the subjected threshold value. The model have the ability to handle large scale dataset with faster response; this is achieved by considering kernel.

The hyperparameters of DTR, LR, RF and SVR are shown in Table 1.

The predicted and initial result obtained by different regression approach for different hotel classes are shown in Table 2. The achieved error value predicts the performance of proposed regression model over the other existing techniques. The predicted result indicate that the proposed architecture has shown better performance on recommendation. The proposed architecture learns the similarity between each items and performed recommendation with high accuracy. The different hotel classes are taken into consideration to show that the proposed architecture is feasible for all kinds of hotels. Moreover, this approach is developed with low cost and low architecture design. Therefore, it can be used by all classes of hotels. The predicted RMS value achieved by 5 – star hotel is found to be, 0.987 (DTR), 1.0 (LR), 0.996 (RF), and 0.992 (SVR) respectively.

The predicted and initial results achieved for 5 star hotel class based on trip type is shown in Table 3. The trip type that are considered in this proposed analysis are Business, couple, family, friend, solo, and Na. Based on these types, the performance metrics like RMS, MSE, MAE, and R-square are evaluated. The evaluated results for 2, 3, 4, and 5 star hotels are shown in Tables 3, 4, 5, and 6. The RMS value achieved by proposed approach for business, couple, family, friends, and Na are found to be 1.09241E−15, 1.01173E−15, 9.93639E−6, 6.03351E−16, and 8.55791E−16 respectively. The obtained values are found better than other existing techniques.

**Table 6:** Accuracy analysis on 2 star hotel class based on trip type (Initial and predicted results)

	Hotel class: 2					
	Trip type	Metrics	Regression models			
			DTR	LR	RF	SVR
Initial	Business	R-Square	0.592695331	0.713202969	0.708197539	0.685679206
		MAE	0.462349095	0.449815572	0.406977742	0.402054757
		MSE	0.5159785	0.36331796	0.369658901	0.398185397
		RMS	0.718316434	0.602758625	0.607995807	0.631019332
	Couple	R-Square	0.303374676	0.53436059	0.521742058	0.534448701
		MAE	0.423722533	0.421966847	0.381803141	0.378503647
		MSE	0.579107661	0.387088066	0.397577907	0.387014819
		RMS	0.760991236	0.622164018	0.630537792	0.622105151
	Family	R-Square	0.677094015	0.715299737	0.745370836	0.745370836
		MAE	0.352732924	0.422769364	0.339042775	0.339042775
		MSE	0.391507732	0.345185161	0.308725422	0.308725422
		RMS	0.625705787	0.587524605	0.555630652	0.555630652
	Friends	R-Square	0.654568507	0.751386727	0.765789877	0.738395989
		MAE	0.394737563	0.410356946	0.355757332	0.362673182
		MSE	0.432844852	0.311526243	0.293478296	0.32780436
		RMS	0.657909456	0.55814536	0.541736371	0.572542016
	Na	R-Square	0.540050043	0.610327148	0.659870709	0.633035718
		MAE	0.455093677	0.499768022	0.408521673	0.436048351
		MSE	0.571445486	0.484132651	0.422579337	0.455919343
		RMS	0.755940134	0.695796415	0.650061026	0.675217997
	Solo	R-Square	0.333439999	0.572021263	0.551740458	0.60279635
		MAE	0.416465505	0.420402539	0.376805692	0.375125808
		MSE	0.682829357	0.438424816	0.459200634	0.406898573
		RMS	0.826334894	0.662136554	0.677643441	0.637886019
Predicted	Business	R-Square	0.965759853	1.0	0.985097791	0.96253946
		MAE	0.081228169	5.76221E−16	0.054558796	0.115794126
		MSE	0.033797671	5.7537E−31	0.014709632	0.036976448
		RMS	0.183841429	7.58532E−16	0.121283271	0.192292611
	Couple	R-Square	0.988419603	1.0	0.988853735	0.97515028
		MAE	0.022399348	7.82466E−16	0.021844711	0.084146211
		MSE	0.006156977	1.22263E−30	0.005926161	0.013211909
		RMS	0.078466407	1.10572E−15	0.076981562	0.11494307
	Family	R-Square	0.96828288	1.0	0.987198206	0.967243981
		MAE	0.053017314	9.81008E−16	0.03587216	0.10303029
		MSE	0.028698699	1.72564E−30	0.011583486	0.029638729
		RMS	0.169406903	1.31364E−15	0.107626606	0.172159023
	Friends	R-Square	0.958664112	1.0	0.984017033	0.969982259
		MAE	0.067327917	1.07044E−15	0.044842833	0.097976718
		MSE	0.037975369	1.43792E−30	0.014683586	0.027577363
		RMS	0.194872699	1.19914E−15	0.121175847	0.166064334
	Na	R-Square	0.935407988	1.0	0.95434319	0.896803043
		MAE	0.07432623	4.40449E−16	0.070653094	0.145032692
		MSE	0.048923705	4.11404E−31	0.034581681	0.078164116
		RMS	0.221187037	6.41408E−16	0.185961503	0.279578461
	Solo	R-Square	0.987618877	1.0	0.992388949	0.896803043
		MAE	0.024559596	6.69498E−16	0.024121313	0.145032692
		MSE	0.008206871	1.18599E−30	0.005045012	0.078164116
		RMS	0.090591781	1.08903E−15	0.071028248	0.279578461

The predicted and initial results achieved for 4 star hotel class based on trip type is shown in Table 4. The performance analysis is performed between the proposed and existing regression models. The models that are taken for comparison are DTR, RF, and SVR. While comparing with other techniques, the proposed approach has shown better performance than other methods. The R-square value achieved by proposed and existing algorithms in solo trip type is found to be 0.971336701 (DTR), 1.0 (LR), 0.99026373 (RF), and 0.985296617 (SVR). However, the predicted RMS performance achieved by LR for Business, couple, family, friends, and solo are found to be 1.80781E−15, 1.37259E−15, 1.91758E−15, 2.29432E−15, and 1.47653E−15 respectively.

The predicted and initial results achieved for 3 star hotel class based on trip type is shown in Table 5. The trip type that are considered in this proposed analysis are Business, couple, family, friend, solo, and Na. Based on these types, the performance metrics like RMS, MSE, MAE, and R-square are evaluated. The predicted RMS value achieved by proposed approach for business, couple, family, friends, Na, and solo are found to be 9.81162E−16, 1.24224E−15, 1.0953E−15, 1.24075E−15, 1.15922E−15, and 1.19088E−15, respectively. The obtained values are found better than other existing techniques.

The predicted and initial results achieved for 2 star hotel class based on trip type is shown in Table 6. The performance analysis is performed between the proposed and existing regression models. The models that are taken for comparison are DTR, RF, and SVR. While comparing with other techniques, the proposed approach has shown better performance than other methods. The R-square value achieved by proposed and existing algorithms in solo trip type is found to be 0.987618877 (DTR), 1.0 (LR), 0.992388949 (RF), and 0.896803043 (SVR). However, the predicted RMS performance achieved by LR for Business, couple, family, friends, Na, and solo are found to be 7.58532E−16, 1.10572E−15, 1.31364E−15, 1.19914E−15, 6.41408E−16, and 1.08903E−15 respectively. The achieved proposed values are found efficient than existing methods.

### Overall rating of different hotel classes on continental, county, and city

**Fig. 2:**
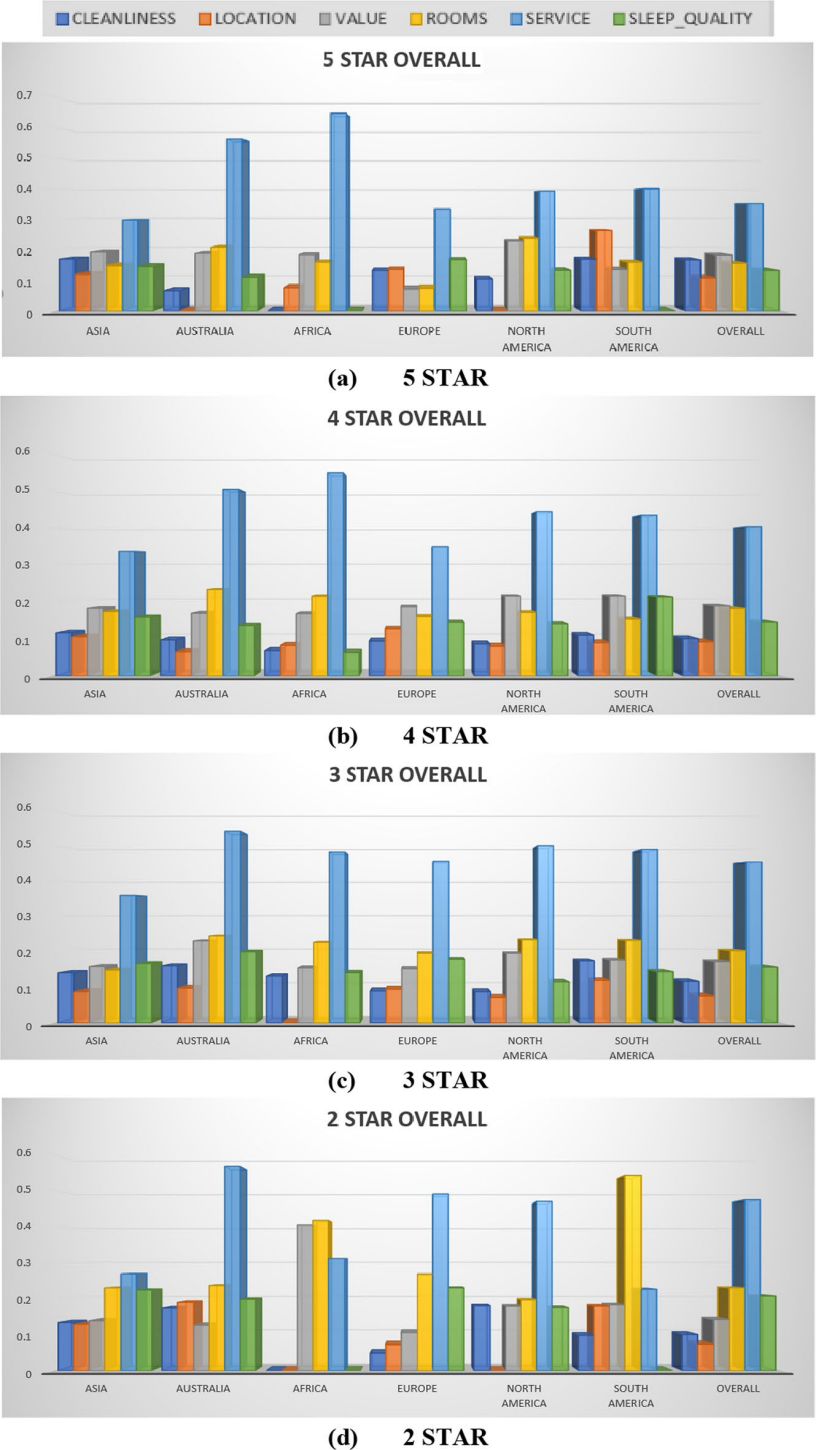
Graphical illustration offor each hotel class upon overall rating on continent. **a** 5 STAR, **b** 4 STAR, **c** 3 STAR, **d** 2 STAR

For each hotel class and trip type, thesignificant contexts were identified upon overall rating on the continental, country, and city to identify the user opinions on hotel stays. Based on this, the overall ratings on the continent (Asia, Australia, Africa, Europe, North America, South America) and country (India, Singapore, Thailand, Germany, US and Brazil), under 5, 4, 3 and 2-star hotels are shown in Table 7 and Fig. 2.

**Table 7:** Final overall results for each hotel class upon country wise

5-STAR OVERALL							
Attribute	India	Singapore	Thailand	Germany	US	Brazil	Overall
Cleanliness	0.1341	NI	NI	0.1442	-0.1001	NI	0.1379
Location	0.1313	NI	NI	NI	NI	NI	0.1084
Value	0.2104	0.3238	0.283	NI	0.1759	0.2457	0.1836
Rooms	0.1834	0.2042	0.3345	0.2373	0.2707	0.2145	0.1555
Service	0.467	0.5753	0.4649	0.6494	0.586	0.5731	0.3517
Sleep quality	0.0955	NI	NI	0.2454	0.1526	0.1982	0.1333
No of Observations	1231	357	216	166	604	202	9215

4-STAR OVERALL							
Attribute	India	Singapore	Thailand	Germany	US	Brazil	Overall
Cleanliness	0.1605	NI	0.068	0.1214	NI	0.1148	0.0999
Location	NI	NI	0.0998	0.085	0.0647	0.1021	0.0914
Value	0.1447	0.314	0.1744	0.2277	0.184	0.155	0.1881
Rooms	0.1505	0.2066	0.2249	0.2148	0.2465	0.2278	0.1812
Service	0.3736	0.5332	0.4255	0.463	0.5805	0.4753	0.4026
Sleep quality	0.2370	NI	0.1915	0.1791	0.1116	0.1805	0.1444
No of Observations	807	739	1002	1132	1953	824	22,575

3-STAR OVERALL							
Attribute	India	Singapore	Thailand	Germany	US	Brazil	Overall
Cleanliness	0.0821	0.2524	NI	0.0942	0.1043	0.1418	0.1165
Location	0.0977		0.1238	0.1285	0.0493	0.0901	0.0752
Value	0.1347	0.3377	0.186	0.1518	0.192	0.1596	0.1713
Rooms	0.1491	0.3026	0.2209	0.1853	0.188	0.2638	0.2022
Service	0.4557	0.5263	0.5526	0.4795	0.5524	0.4838	0.4524
Sleep quality	NI	NI	0.2824	0.2321	0.1271	0.1391	0.1564
No of Observations	1549	83	398	760	2960	1189	22,827

2-STAR OVERALL							
Attribute	India	Singapore	Thailand	Germany	US	Brazil	Overall
Cleanliness	NI	NI	NI	NI	0.1933	0.1356	0.0996
Location	0.2535	NI	0.181	NI	0.0811	NI	0.0735
Value	0.1685	NI	NI	0.2016	0.1794	0.1645	0.1415
Rooms	NI	NI	NI	0.2392	0.1935	0.2798	0.2302
Service	0.1671	NI	0.2534	0.6263	0.4826	0.4411	0.4739
Sleep quality	NI	NI	NI	NI	0.1130	0.2123	0.2075
No of Observations	185	NI	122	93	910	232	5136

The overall rating achieved by 2, 3, 4, and 5 star hotels of different continents is analysed and the result is shown in Fig. 2. In this method, the Asia, Australia, Africa, Europe, North America, and South America continents are considered for analysis. Among all these, the 5-star hotels of Africa continent has attained high ratings than all other hotels in remaining continents. The service attribute of 2-star and 3-star hotels in Australia has attained higher ratings than other 2-star hotels of remaining continents. In all continents, the 3-star and 4-star hotels have attained almost satisfactory ratings. Africa continent has obtained ‘0’ rating for cleanliness and location attributes. However, the cleanliness of 5-star hotels of all continent have received few ratings, which describe that the 5-star hotels are showing huge importance to cleanliness. These attributes are analysed by proposed regression model to recommend the highly rated hotels to customers based on the hotel classes.

The country-wise hotel ratings achieved by 2, 3, 4, and 5 star hotels based on hotel attributes are shown in Table 7. The countries that are taken for analysis are India, Singapore, Thailand, Germany, US, and Brazil. Finally, the overall ratings achieved by each countries for various attributes is also determined. The attributes that are taken into consideration for hotel recommendation are cleanliness, location, value, rooms, service, and sleep quality. The attributes that are taken for analysis during recommendation is mandatory to help the customers to select best hotels during trips. In case if any of the attributes are not proper means the hotel recommendation rating will get reduced. Therefore, to guide the customers in proper area the ratings provided by all customers related to the visited hotels is mandatory to improve the recommendation process.

### Overall ratings for different trip types under 5-star hotel class

**Fig. 3:**
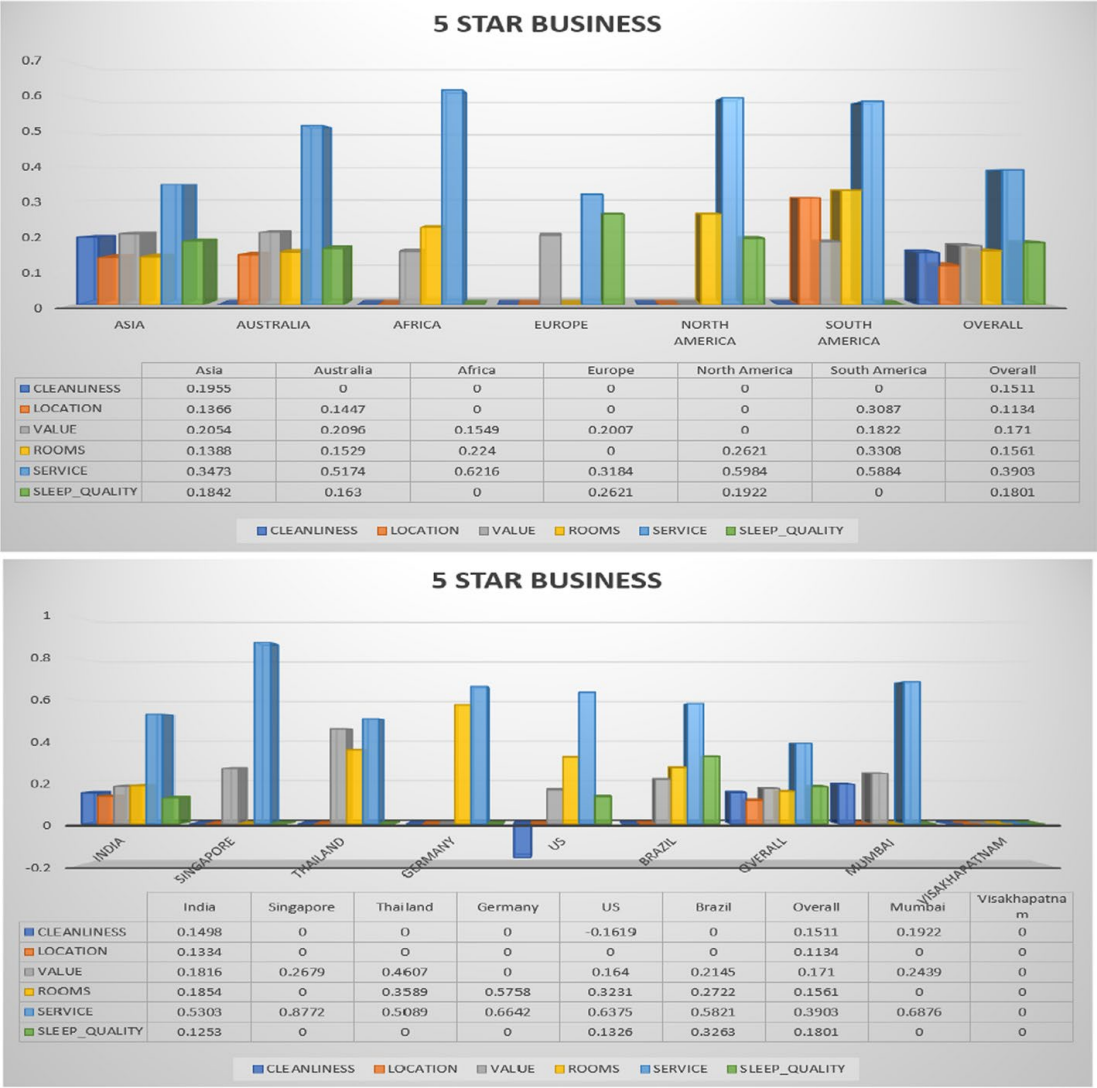
Hotel class: 5 star, Trip Type: Travelled on business (continent, country and cities)

**Fig. 4:**
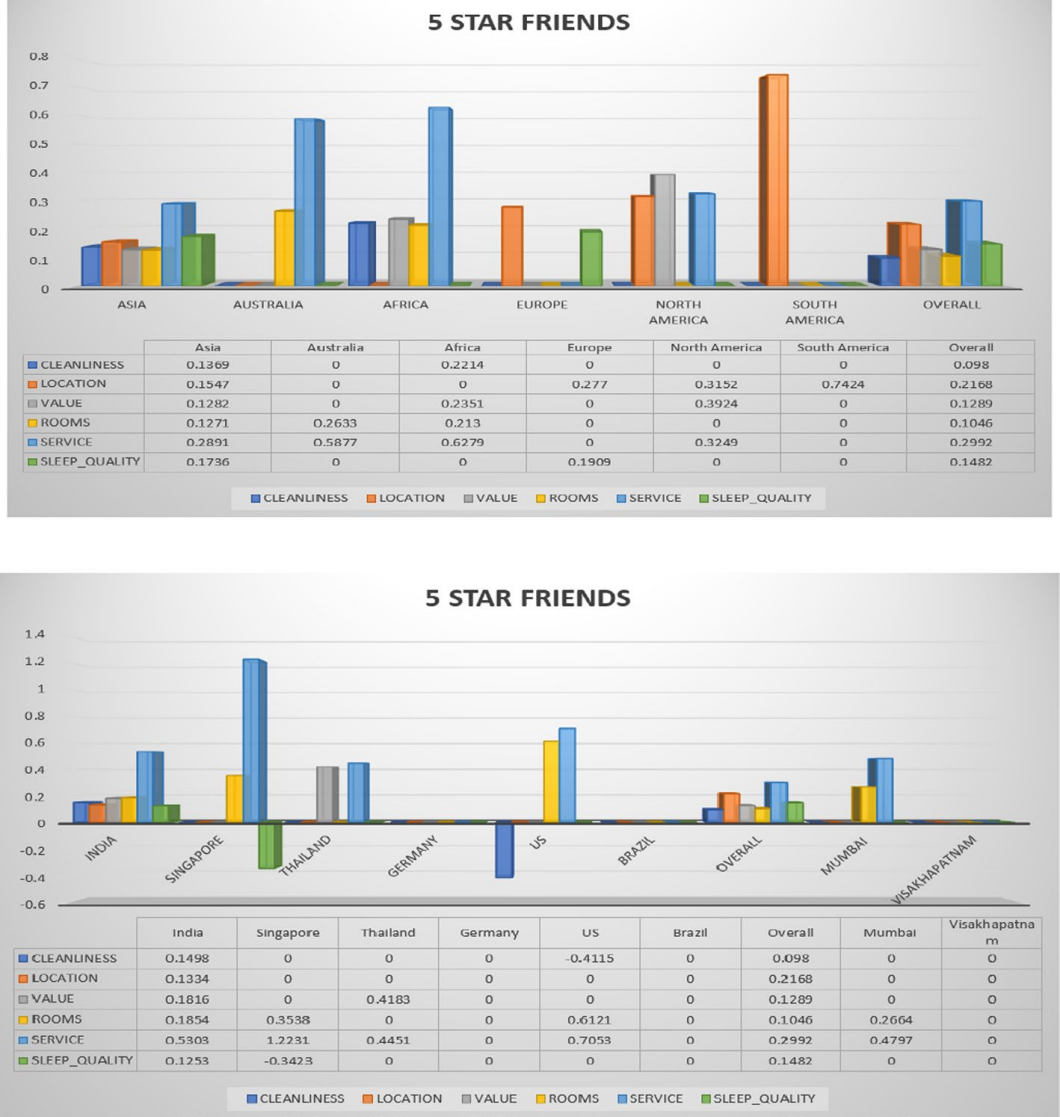
Hotel class: 5 star, Trip Type: Travelled with friends (continent, country and cities)

**Fig. 5:**
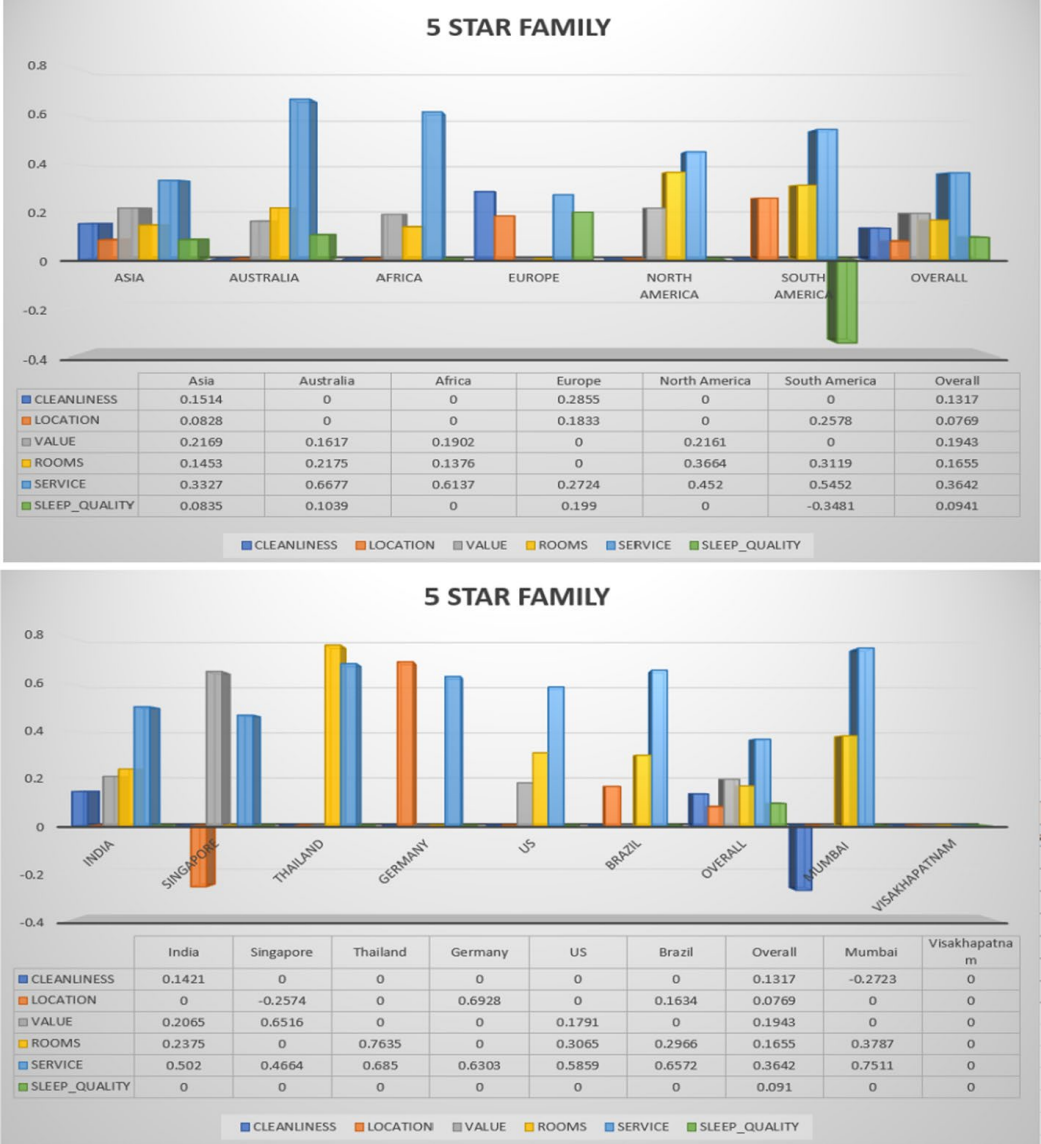
Hotel class: 5 star, Trip Type: Travelled with family (continent, country and cities)

**Fig. 6:**
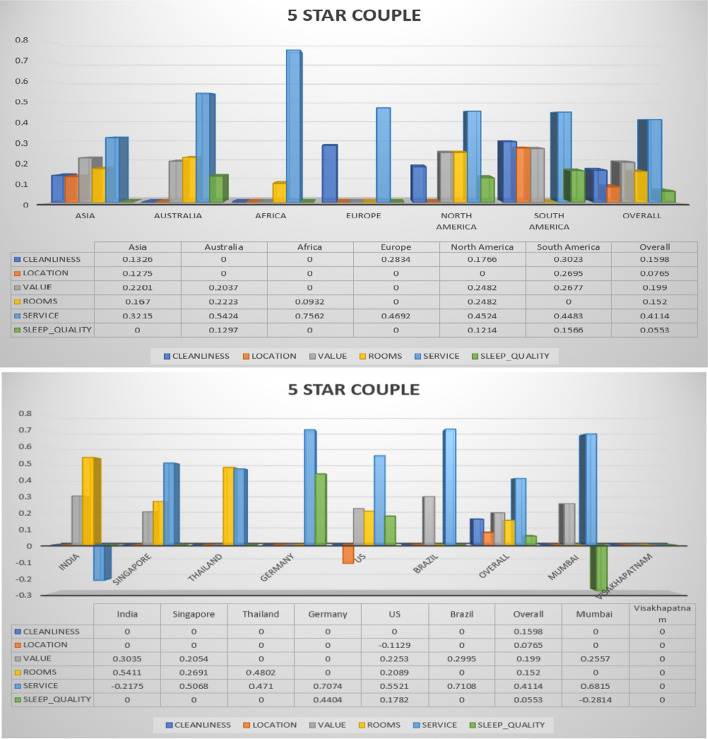
Hotel class: 5 star, Trip Type: Travelled as a couple (continent, country and cities)

**Fig. 7:**
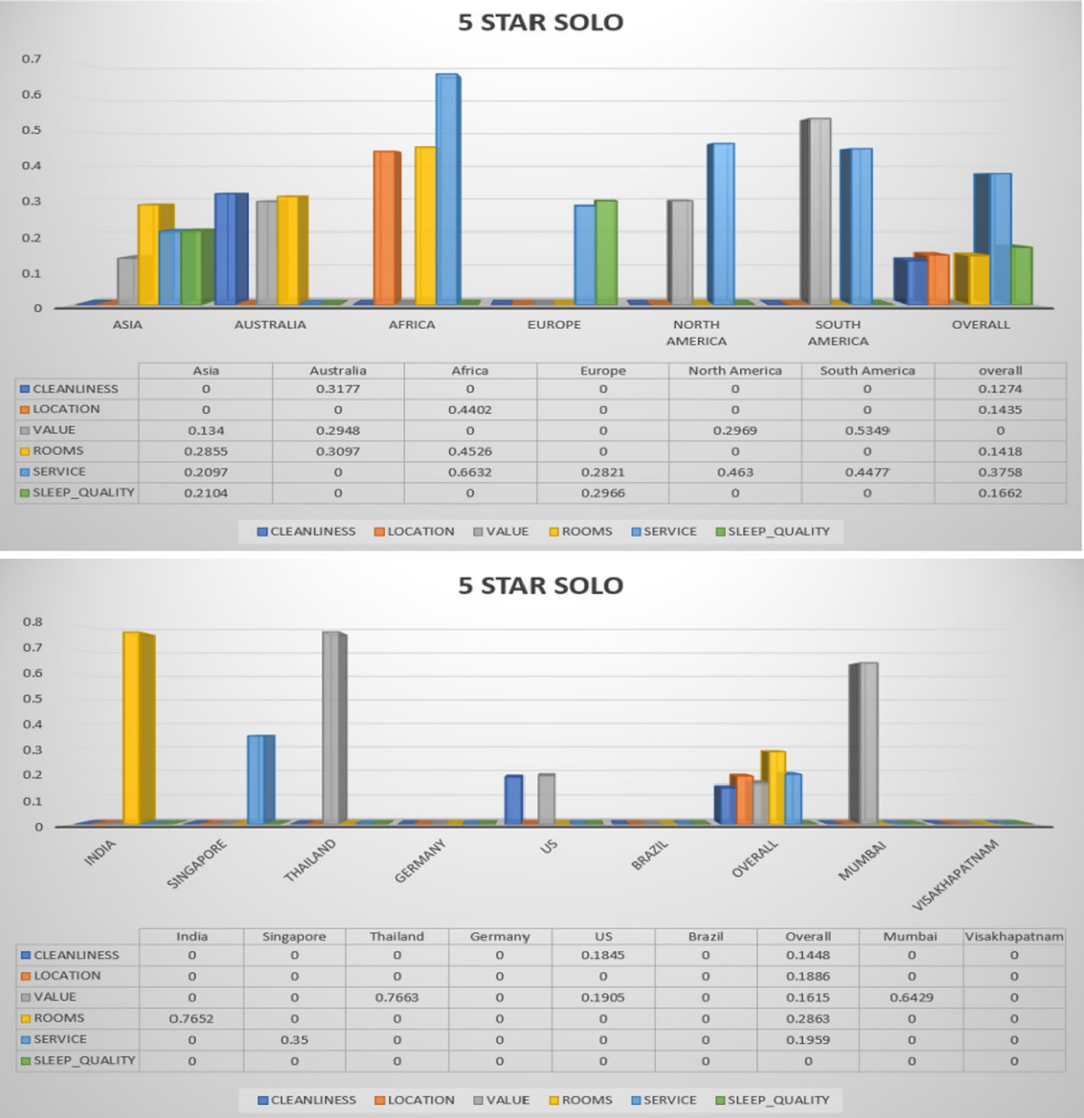
Hotel class: 5 stars, Trip Type: Travelled as a solo (continent, country and cities)

The following Figs. 3, 4, 5, 6, 7 describe different trip types’ ratings on five-star hotel classes. The ratings of different significant contexts of continents, countries and cities are illustrated graphically. Based on the ranking in the below figures, the 5-star hotel class users under business trip type give first more importance to cleanliness. The figures below show the results on different trip types in the same star hotel.

The overall ratings achieved by different continent, countries, and cities for hotel class 5 and trip type (business) is shown in Figure 3. The business class peoples normally held important meetings in 5-star hotels. They plan to conduct meetings in 5-star hotels by spending huge amount. Therefore it is mandatory to maintain better cleanliness, rooms, services, sleep quality, and values. These suggested features needs to be better in all 5-star hotels to recommend the hotels to customers. For that, the reviews provided by customers is very much useful. These reviews are analysed by recommendation algorithms, based on the similarity analysis the hotel recommendation will happen. The service quality provided to all business people from India, Singapore, and US are found best than other countries. The 5-star hotels from such countries are highly recommended to all business running customers to conduct a standard and perfect official meetings.

The overall ratings achieved by different continent, countries, and cities for hotel class 5 and trip type (friends) is shown in Fig. 4. The countries that are taken for comparison are India, Singapore, Thailand, US, and Brazil. Finally, the overall performance is also described. The cities that are considered for performance analysis are Mumbai, and Vishakhapatnam (India). The parameters that are considered for performance analysis from different hotel class data are cleanliness, location, value, rooms, service, and sleep quality. These are the parameters considered in this work for rating and recommending the hotels. These all parameters needs to be satisfied by all classes of hotels, therefore such hotel can be recommended to number of users based on ratings.

The overall ratings achieved by different continent, countries, and cities for hotel class 5 and trip type (family) is shown in Fig. 5. The family normally expect high standard hotels and rooms, because the family is a group of children, aged persons, and health affected persons. Therefore it is mandatory to maintain better cleanliness, rooms, services, sleep quality, and values. These suggested features needs to be better in all hotels to recommend the hotels to customers. For that, the reviews provided by customers is very much useful. These reviews are analysed by recommendation algorithms, based on the similarity analysis the hotel recommendation will happen. The recommendation analysis is separate for all hotel classes. The overall rating provided by families for 5 star hotel in Vishakhapatnam is 0. This rating may degrade the recommendation of such hotels for customers. Rooms in Thailand hotels has attained high ratings than other countries, this rating makes the Thailand hotel popular among all family. This analysis has shown that the hotels in US (Country), North America (Continent), and Mumbai (city) are highly recommended to trip planning families.

The overall ratings achieved by different continent, countries, and cities for hotel class 5 and trip type (couple) is shown in Fig. 6. The couples normally expect high standard hotels and rooms, therefore it is mandatory to improve the quality of hotels. For that, the reviews provided by customers is very much useful. These reviews are analysed by recommendation algorithms, based on the similarity analysis the hotel recommendation will happen. The recommendation analysis is separate for all hotel classes. The overall rating provided by couple for 5 star hotel in Vishakhapatnam is 0. This rating may degrade the recommendation of such hotels for customers. For continent-wise comparison, the Europe has obtained less ratings than other continents. This analysis has shown that the hotels in US (Country), North America (Continent), and Mumbai (city) are highly recommended to trip planning customers.

The overall ratings achieved by different continent, countries, and cities for hotel class 5 and trip type (solo) is shown in Fig. 7. The countries that are taken for comparison are India, Singapore, Thailand, US, and Brazil. Finally, the overall performance is also described. The cities that are considered for performance analysis are Mumbai, and Vishakhapatnam (India). The parameters that are considered for performance analysis from different hotel class data are cleanliness, location, value, rooms, service, and sleep quality. These are the parameters considered in this work for rating and recommending the hotels. These all parameters needs to be satisfied by all classes of hotels, therefore such hotel can be recommended to number of users based on ratings.

### ANOVA (Analysis of variance) test

The prime aspect of this research is to focus on trip types of users to analyse the significant context with various hotel classes. The ANOVA results of different hotel classes are shown in (Table 8). Also, the ANOVA results of different trip types under different hotels are shown in (Tables 9, 10, 11, 12). By performing ANOVA tests, significant differences between each predictors with overall user ratings are identified for each type of trip. Also, the multi-collinearity test is performed between independent variables to identify the collinearity between them. If the value of VIF lies 1–10, there will be no multi-collinearity, and if the VIF factor is < 1 or  > 10, the multi-collinearity occurs.

**Table 8:** Analysis of Variance (ANOVA) based on different hotel classes

Hotel Class	Model	Df	Sum of square	Mean Square	F value	Pr (> F)	Remark	VIF
5 Star	SERVICE	1	5238.4	5238.4	9381.373	< 2.2e−16	significant	1.701318
	value	1	564	564	1010.144	< 2.2e−16	significant	1.581975
	Rooms	1	288.2	288.2	516.133	< 2.2e−16	significant	1.659956
	Sleep-Quality	1	114.2	114.2	204.605	< 2.2e−16	significant	1.540122
	Cleanliness	1	92.7	92.7	165.997	< 2.2e−16	significant	1.597566
	Location	1	20.4	20.4	36.997	1.57E−09	significant	1.406474
	Residuals	8074	4493.3	0.6	NI	NI	NI	NI
4 Star	SERVICE	1	11,050.6	11,050.6	29,370.99	< 2.2e−16	significant	1.734618
	value	1	1106.4	1106.4	2940.69	< 2.2e−16	significant	1.645533
	Rooms	1	689.1	689.1	1831.49	< 2.2e−16	significant	1.650091
	Sleep-Quality	1	254	254	675.02	< 2.2e−16	significant	1.511012
	Cleanliness	1	81.2	81.2	215.92	< 2.2e−16	significant	1.52539
	Location	1	49.7	49.7	132.19	< 2.2e−16	significant	1.262507
	Residuals	21,310	8017.7	0.4	NI	NI	NI	NI
3 Star	SERVICE	1	11,851.9	11,851.9	36,862.79	< 2.2e−16	significant	1.785064
	value	1	739.5	739.5	2300.18	< 2.2e−16	significant	1.545586
	Rooms	1	661.4	661.4	2057.04	< 2.2e−16	significant	1.562195
	Sleep-Quality	1	236.4	236.4	735.29	< 2.2e−16	significant	1.419153
	Cleanliness	1	106.2	106.2	330.35	< 2.2e−16	significant	1.444825
	Location	1	44.8	44.8	139.48	< 2.2e−16	significant	1.216742
	Residuals	21,620	6951.1	0.3	NI	NI	NI	NI
2 Star	SERVICE	1	3182.2	3182.2	10,676.73	< 2.2e−16	significant	1.82484
	value	1	196	196	657.477	< 2.2e−16	significant	1.538713
	Rooms	1	228.8	228.8	767.597	< 2.2e−16	significant	1.612716
	Sleep-Quality	1	85.4	85.4	586.368	< 2.2e−16	significant	1.435954
	Cleanliness	1	25.1	25.1	84.199	< 2.2e−16	significant	1.497699
	Location	1	14.9	14.9	49.831	1.90E−12	significant	1.218268
	Residuals	5139	1531.7	0.3	NI	NI	NI	NI

**Table 9:** Analysis of Variance of 5 star hotel class based on trip type

5 STAR HOTEL								
Trip Type	Model	Df	Sum of square	Mean Square	F value	Pr(> F)	Remark	vif
Business	SERVICE	1	1809.55	1809.55	3210.868	< 2.2e−16	significant	1.680785
	value	1	232.47	232.47	412.491	< 2.2e−16	significant	1.718335
	Rooms	1	97.84	97.84	173.615	< 2.2e−16	significant	1.783331
	Sleep-Quality	1	55.97	55.97	99.307	< 2.2e−16	significant	1.63776
	Cleanliness	1	32.8	32.8	58.207	3.42E−14	significant	1.749201
	Location	1	8.93	8.93	15.85	7.07E−05	significant	1.416789
	Residuals	5139	1309.18	0.56	NI	NI	NI	NI
Couple	SERVICE	1	1152.52	1152.52	2634.7167	< 2.2e−16	significant	1.649211
	value	1	118.52	118.52	270.9755	< 2.2e−16	significant	1.432812
	Rooms	1	45.62	45.62	104.2928	< 2.2e−16	significant	1.531331
	Sleep-Quality	1	5.08	5.08	11.6103	0.0006666	significant	1.391232
	Cleanliness	1	22.54	22.54	51.5368	9.31E−13	significant	1.454192
	Location	1	0.78	0.78	1.7788	0.1824216	insignificant	1.32593
	Residuals	2417	1057.28	0.44	NI	NI	NI	NI
Family	SERVICE	1	1205.45	1205.45	2589.4634	< 2.2e−16	significant	1.921574
	value	1	129.42	129.42	278.011	< 2.2e−16	significant	1.917425
	Rooms	1	70.69	70.69	151.8553	< 2.2e−16	significant	1.945198
	Sleep-Quality	1	19.13	19.13	41.0875	1.87E−10	significant	1.639531
	Cleanliness	1	5.63	5.63	12.0972	5.18E−04	significant	1.690062
	Location	1	0.46	0.46	0.9789	0.3226074	insignificant	1.536636
	Residuals	1730	805.35	0.47	NI	NI	NI	NI
Friends	SERVICE	1	485.66	485.66	626.87	< 2.2e−16	significant	1.488284
	value	1	60.48	60.48	78.065	< 2.2e−16	significant	1.429711
	Rooms	1	29.23	29.23	37.723	1.33E−09	significant	1.404327
	Sleep-Quality	1	10.28	10.28	13.264	0.0002895	significant	1.428758
	Cleanliness	1	12	12	15.485	9.10E−05	significant	1.564381
	Location	1	10.05	10.05	12.973	0.0003372	significant	1.415283
	Residuals	738	571.76	0.77	NI	NI	NI	NI
NA	SERVICE	1	181.915	181.915	215.915	< 2.2e−16	significant	1.606145
	value	1	13.173	13.173	15.586	9.62E−05	significant	1.326224
	Rooms	1	18.549	18.549	21.9473	4.09E−06	significant	1.388735
	Sleep-Quality	1	25.498	25.498	30.1699	7.87E−08	significant	1.425978
	Cleanliness	1	11.025	11.025	13.0452	3.51E−04	significant	1.484224
	Location	1	0.579	0.579	0.6846	0.4085877	insignificant	1.312089
	Residuals	333	281.435	0.845	NI	NI	NI	NI
Solo	SERVICE	1	251.12	251.118	296.9813	< 2.2e−16	significant	1.633852
	value	1	10.57	10.567	12.4967	0.0004479	significant	1.348226
	Rooms	1	19.54	19.538	23.1061	2.07E−06	significant	1.496791
	Sleep-Quality	1	13.02	13.021	15.3993	1.00E−04	significant	1.498829
	Cleanliness	1	8.33	8.326	9.8471	0.0018076	significant	1.329458
	Location	1	7.7	7.695	9.1004	0.0026938	significant	1.339166
	Residuals	471	398.26	0.846	NI	NI	NI	NI

**Table 10:** Analysis of Variance of 4 star hotel class based on trip type

4 STAR HOTEL								
Trip Type	Model	Df	Sum of square	Mean Square	F value	Pr(> F)	Remark	vif
Business	SERVICE	1	2941.33	2941.33	7319.049	< 2.2e−16	significant	1.74422
	value	1	391.85	391.85	975.068	< 2.2e−16	significant	1.821714
	Rooms	1	174.76	174.76	434.855	< 2.2e−16	significant	1.789271
	Sleep-Quality	1	72.6	72.6	180.661	< 2.2e−16	significant	1.676617
	Cleanliness	1	36.31	36.31	90.35	< 2.2e−16	significant	1.645338
	Location	1	7.47	7.47	18.586	1.66E−05	significant	1.32015
	Residuals	4541	1824.9	0.4	NI	NI	NI	NI
Couple	SERVICE	1	2772.36	2772.36	8542.342	< 2.2e−16	significant	1.707135
	value	1	306.84	306.84	912.251	< 2.2e−16	significant	1.50884
	Rooms	1	160.65	160.65	477.607	< 2.2e−16	significant	1.53595
	Sleep-Quality	1	63.95	63.95	190.113	< 2.2e−16	significant	1.377149
	Cleanliness	1	13.38	13.38	39.766	3.04E−10	significant	1.438046
	Location	1	12.64	12.64	37.574	9.28E−10	significant	1.193221
	Residuals	6958	2340.36	0.34	NI	NI	NI	NI
Family	SERVICE	1	2684.74	2684.74	7400.904	< 2.2e−16	significant	1.774843
	value	1	229.29	229.29	632.076	< 2.2e−16	significant	1.764872
	Rooms	1	204.79	204.79	564.539	< 2.2e−16	significant	1.75737
	Sleep-Quality	1	40.44	40.44	111.486	< 2.2e−16	significant	1.618987
	Cleanliness	1	17.3	17.3	47.693	5.60E−12	significant	1.610035
	Location	1	4.92	4.92	13.572	2.32E−04	significant	1.315588
	Residuals	5047	1830.84	0.36	NI	NI	NI	NI
Friends	SERVICE	1	963.26	963.26	2348.431	< 2.2e−16	significant	1.701396
	value	1	74.75	74.75	182.238	< 2.2e−16	significant	1.638321
	Rooms	1	50.34	50.34	122.718	< 2.2e−16	significant	1.520493
	Sleep-Quality	1	17.52	17.52	42.706	7.94E−11	significant	1.452488
	Cleanliness	1	19.52	19.52	47.588	6.92E−12	significant	1.457153
	Location	1	7.38	7.38	18.002	2.30E−05	significant	1.234531
	Residuals	2122	870.38	0.41	NI	NI	NI	NI
NA	SERVICE	1	717.02	717.02	1717.5282	< 2.2e−16	significant	1.734495
	value	1	52.87	52.87	126.644	< 2.2e−16	significant	1.433546
	Rooms	1	41.31	41.31	98.9591	< 2.2e−16	significant	1.531309
	Sleep-Quality	1	19.31	19.31	47.4751	9.76E−12	significant	1.382249
	Cleanliness	1	3.67	3.67	8.7984	0.003086	significant	1.398805
	Location	1	9.09	9.09	21.7689	3.49E−06	significant	1.225912
	Residuals	1015	423.73	0.42	NI	NI	NI	NI
Solo	SERVICE	1	862.22	862.22	2180.854	< 2.2e−16	significant	1.742013
	value	1	59.11	59.11	149.5127	< 2.2e−16	significant	1.592201
	Rooms	1	56.92	56.92	143.959	< 2.2e−16	significant	1.684333
	Sleep-Quality	1	36.97	36.97	93.5204	< 2.2e−16	significant	1.499656
	Cleanliness	1	0.71	0.71	1.8009	0.1798	significant	1.487551
	Location	1	14.84	14.84	37.5469	1.12E−09	significant	1.2859
	Residuals	1592	629.41	0.4	NI	NI	NI	NI

**Table 11:** Analysis of Variance of 3 star hotel class based on trip type

3 STAR HOTEL								
Trip Type	Model	Df	Sum of square	Mean Square	F value	Pr(> F)	Remark	vif
Business	SERVICE	1	2379.71	2379.71	6825.1	< 2.2e−16	significant	1.774596
	value	1	184.82	184.82	530.07	< 2.2e−16	significant	1.681829
	Rooms	1	150.87	150.87	432.71	< 2.2e−16	significant	1.694853
	Sleep-Quality	1	60.63	60.63	173.71	< 2.2e−16	significant	1.543683
	Cleanliness	1	23.72	23.72	68.02	< 2.2e−16	significant	1.528505
	Location	1	3.94	3.94	11.31	0.0007788	significant	1.241848
	Residuals	3556	1239.87	0.35	NI	NI	NI	NI
Couple	SERVICE	1	3260.8	3260.8	11,534.987	< 2.2e−16	significant	1.756649
	value	1	208.9	208.9	738.929	< 2.2e−16	significant	1.422995
	Rooms	1	193.5	193.5	684.416	< 2.2e−16	significant	1.436299
	Sleep-Quality	1	70.4	70.4	249.068	< 2.2e−16	significant	1.351903
	Cleanliness	1	44.5	44.5	157.425	< 2.2e−16	significant	1.359848
	Location	1	13.3	13.3	47.143	7.15E−12	significant	1.170638
	Residuals	3556	2035.1	0.3	NI	NI	NI	NI
Family	SERVICE	1	2939.02	2939.02	8819.45	< 2.2e−16	significant	1.800058
	value	1	179.49	179.49	538.618	< 2.2e−16	significant	1.670787
	Rooms	1	120.2	120.2	360.692	< 2.2e−16	significant	1.706803
	Sleep-Quality	1	41.8	41.8	125.438	< 2.2e−16	significant	1.526381
	Cleanliness	1	14.39	14.39	43.182	5.52E−11	significant	1.52387
	Location	1	4.61	4.61	13.834	2.02E−04	significant	1.252643
	Residuals	4791	1596.57	0.33	NI	NI	NI	NI
Friends	SERVICE	1	1153.94	1153.94	3235.079	< 2.2e−16	significant	1.794296
	value	1	41.97	41.97	117.661	< 2.2e−16	significant	1.43645
	Rooms	1	101.73	101.73	285.211	< 2.2e−16	significant	1.440246
	Sleep-Quality	1	31.96	31.96	89.612	< 2.2e−16	significant	1.398929
	Cleanliness	1	14.43	14.43	40.453	2.38E−10	significant	1.370722
	Location	1	15.07	15.07	42.26	9.60E−11	significant	1.211129
	Residuals	2519	898.52	0.36	NI	NI	NI	NI
NA	SERVICE	1	900.48	900.48	3128.921	< 2.2e−16	significant	1.998244
	value	1	46.86	46.86	162.831	< 2.2e−16	significant	1.737501
	Rooms	1	35.53	35.53	123.446	< 2.2e−16	significant	1.696424
	Sleep-Quality	1	9.82	9.82	34.875	6.42E−09	significant	1.259639
	Cleanliness	1	10.32	10.32	35.875	2.69E−09	significant	1.554381
	Location	1	12.29	12.29	42.71	8.96E−11	significant	1.27069
	Residuals	1359	391.11	0.29	NI	NI	NI	NI
Solo	SERVICE	1	1039.88	1039.88	3136.331	< 2.2e−16	significant	1.708732
	value	1	81.35	81.35	245.358	< 2.2e−16	significant	1.444136
	Rooms	1	60.49	60.49	182.444	< 2.2e−16	significant	1.527846
	Sleep-Quality	1	26.49	26.49	78.491	< 2.2e−16	significant	1.382859
	Cleanliness	1	26.02	26.02	13.638	2.27E−04	significant	1.443302
	Location	1	4.52	4.52	19.87	8.72E−06	significant	1.23539
	Residuals	2161	716.5	0.33	NI	NI	NI	NI

**Table 12:** Analysis of Variance of 2 star hotel class based on trip type

2 STAR HOTEL								
Trip Type	Model	Df	Sum of square	Mean Square	F value	Pr(> F)	Remark	vif
Business	SERVICE	1	340.15	340.15	1103.1703	< 2.2e−16	significant	1.853245
	value	1	61.75	61.75	200.262	< 2.2e−16	significant	1.968704
	Rooms	1	27.48	27.48	89.1177	< 2.2e−16	significant	2.139497
	Sleep-Quality	1	8.31	8.31	26.9433	3.16E−07	significant	1.97294
	Cleanliness	1	11.53	11.53	37.3937	2.08E−09	significant	1.814568
	Location	1	0.94	0.94	3.0479	0.08152	insignificant	1.394531
	Residuals	455	140.3	0.31	NI	NI	NI	NI
Couple	SERVICE	1	675.27	675.27	2144.5952	< 2.2e−16	significant	1.787364
	value	1	34.44	34.44	109.3642	< 2.2e−16	significant	1.455918
	Rooms	1	76.13	76.13	241.796	< 2.2e−16	significant	1.549944
	Sleep-Quality	1	36.74	36.74	116.6861	< 2.2e−16	significant	1.414444
	Cleanliness	1	2.26	2.26	7.1854	0.007429	significant	1.496427
	Location	1	4.89	4.89	15.5299	8.49E−05	significant	1.189579
	Residuals	1534	483.01	0.31	NI	NI	NI	NI
Family	SERVICE	1	744.96	744.96	2645.492	< 2.2e−16	significant	1.762328
	value	1	44.3	44.3	157.309	< 2.2e−16	significant	1.731201
	Rooms	1	41.13	41.13	146.06	< 2.2e−16	significant	1.725919
	Sleep-Quality	1	14.58	14.58	51.787	1.27E−12	significant	1.636601
	Cleanliness	1	8.57	8.57	30.426	4.49E−08	significant	1.698072
	Location	1	4.09	4.09	14.524	0.0001474	significant	1.346154
	Residuals	937	263.86	0.28	NI	NI	NI	NI
Friends	SERVICE	1	619.66	619.66	2293.756	< 2.2e−16	significant	1.991383
	value	1	20.31	20.31	75.18	< 2.2e−16	significant	1.461245
	Rooms	1	26.62	26.62	98.551	< 2.2e−16	significant	1.515202
	Sleep-Quality	1	9.75	9.75	36.074	2.89E−09	significant	1.314468
	Cleanliness	1	5.22	5.22	19.327	1.25E−05	significant	1.420817
	Location	1	1.98	1.98	7.327	0.006938	significant	1.152492
	Residuals	797	215.31	0.27	NI	NI	NI	NI
NA	SERVICE	1	245.253	245.253	912.1098	< 2.2e−16	significant	2.119269
	value	1	15.551	15.551	57.8339	2.48E−13	significant	1.79915
	Rooms	1	17.171	17.171	63.8619	1.81E−14	significant	1.871509
	Sleep-Quality	1	2.398	2.398	8.9189	0.0030144	significant	1.315677
	Cleanliness	1	2.717	2.717	10.1052	0.0016059	significant	1.576093
	Location	1	3.108	3.108	11.5572	0.0007501	significant	1.3109
	Residuals	362	97.336	0.269	NI	NI	NI	NI
Solo	SERVICE	1	549.98	549.98	1965.9078	< 2.2e−16	significant	1.959543
	value	1	26.69	26.69	95.3905	< 2.2e−16	significant	1.418681
	Rooms	1	39.41	39.41	140.8822	< 2.2e−16	significant	1.464994
	Sleep-Quality	1	17.76	17.76	63.4852	4.30E−15	significant	1.343314
	Cleanliness	1	1.02	1.02	3.6439	0.056556	insignificant	1.34127
	Location	1	2.01	2.01	7.1829	0.007479	significant	1.143535
	Residuals	1019	285.07	0.28	NI	NI	NI	NI

*NI* no instance

The ANOVA analysis for 2, 3, 4, and 5 star hotels under different trip type is shown in (Tables 9, 10, 11, 12). These tables are provided to statistically analyse the performance of proposed recommendation system. The ANOVA analysis result shown by proposed approach for different trip type is found better and efficient. This analysis is carried out by considering different qualifying parameters, they are service, rooms, sleep-quality, location, and residuals. Based on these metrics, the statistical analysis is performed. Most of the parameters have shown significant value whereas few have come under NI. This analysis has conveyed the efficiency of proposed approach in efficient manner. Normally, the regression approaches are found efficient for similarity based processing. Regression approaches statistically analyse all data and perform the recommendation in perfect manner. Due to this merit, the regression model is introduced in this work and has attained better recommendation result with less error rate. This approach mainly concentrates on improving the recommendation accuracy by considering the error rate, however it fails to consider the cold-start and data sparsity issues.

### Comparative analysis between proposed and existing techniques:

**Fig. 8:**
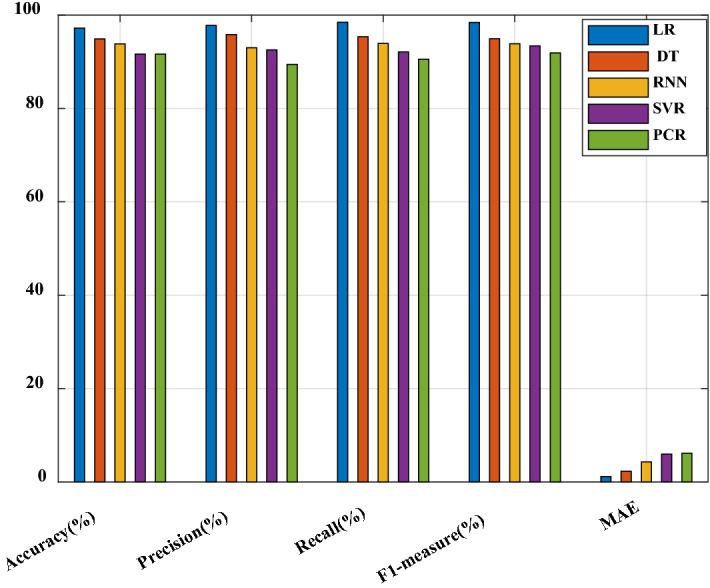
Performance comparison between proposed and existing techniques

The comparative analysis for proposed and existing hotel recommendation architecture is explained in below section. The accuracy, precision, recall, f1-score, and MAE comparison is shown in Fig. 8.

The accuracy, precision, recall, f1-score, and MAE of proposed is compared with existing techniques to show the efficiency of proposed recommendation algorithm. The proposed algorithm analyses the cleanliness, service, value, room-quality, value attributes to perform the efficient recommendation. Using LR the recommendation process is performed, before that the similarity analysis is carried out which enhances the performances of proposed approach. The techniques that are taken for comparison are SVR, DT (decision tree), RNN (Recurrent neural network), and PCR (Principal component regression). Among all these techniques, the proposed architecture has achieved efficient performance.

The performance comparison between proposed and existing techniques is shown in Table 13. The performance of proposed architecture is found better than other existing techniques. The MAE of proposed is 0.068, whereas the MAE of DNN is 0.46. This comparison illustrates the efficiency of proposed regression technique. This is because the regression models will show efficient performance in analysing the statistical values and achieve a better performance. Due to this advantage, the LR regression model is introduced which has also attained an efficient performance in recommendation.

**Table 13:** Comparison between proposed and existing techniques

Ref no.	Technique	Dataset	Performance metrics
Proposed	MRBE	Tripadvisor	MAE–0.0689 Precision–0.968 Recall–0.935 F1-score–0.95
[16]	HOSVD	Tripadvisor	MAE–0.723
[17]	ANFIS	Tripadvisor	Recall–0.84 F1-score–0.839 Precision–0.818
[18]	AEMC	Yahoo, Movies and Tripadvisor dataset	MAE–0.64 RMSE–0.72
[19]	DNN (Deep neural network)	BeerAdvocate website	MAE–0.4616 Recall–0.5284 Precision–0.8559 F1-score–0.6517
[22]	BERT	Tripadvisor	NDCG @15–0.569 NDCG @10–0.606 NDCG @5–0.694
[23]	kNN (k-nearest neighbor)	MovieLens and Film trust	MAE–0.18 Standard deviation (SD)–1.39
[1]	SOM	Tripadvisor	Precision–0.948 F1-score–0.934 MAE–0.753
[26]	Fitting trust algorithm	MovieLens dataset	MAE–0.7

## Conclusion

Multi-Criteria travel recommender systems represent ratings of user views for different contextual segments. However, since user preferences vary from one another on tourism hotel stays due to their dynamic behaviors. It is a big challenge for online travel recommenders to judge accurate predictions of users. Moreover, due to sparsity and the curse of dimensionality, these recommenders still face many problems in generating accurate recommendations for every user since the user is interested in only a few segments. In this research the multi-criteria recommender algorithm is introduced to recommend hotels upon hotel classes and trip types. Initially the data was extracted from the Trip Advisor across different continents, countries and cities. The second stage is data pre-processing. The item-item-collaborative approach using Adjusted Cosine Similarity is introduced for the replacement of missing values. The multi regression backward elimination is introduced to analyse the impact of contextual segments on the overall rating. Here, ordinary least squares (O.L.S) regression model is designed to reduce the residual sum of squares. To identify the significant context checking multi collinearity among the independent variable is essential and this can be processed with respect to variance impact factor (VIF). In the experimental scenario the performance measure of R-square, MAE, MSE and RMSE are evaluated under several regression techniques. The results can be analysed under both the hotel (2, 3, 4 and 5 star) and trip-type (Business, Family, Friends, Couple, Solo, N.A) under continent and country wise. In this research the scalability issue of the multi criteria system had not examined and in future it will be conducted using an efficient algorithm. Along with that, few additional metrics will also evaluated in future to determine the efficiency of architecture in recommendation system.

## Data Availability

The dataset was collected from Trip Advisor through web scrapping from 93 cities across the world from Six continental tourism cities Asia, Europe, North-America, South-America, Africa and Australia.

## Abbreviations

VIF: Variance inflation factor
ANOVA: Analysis of Variance
MAE: Mean absolute error
MSE: Mean squared error
RMSE: Root mean squared error
CF: Collaborative filtering
MCRS: Multi criteria recommender systems
HOSVD: Higher order singular value decompositon
SOM: Self-organizing map
ANFIS: Adaptive neuro-fuzzy inference systems
BERT: Bidirectional encoder representations form transformers
HR: Hit ratio
NDCG: Normalized discounted cumulative gain
MRBE: Multiple regression backward elimination
